# Extraordinary linear dynamic range in laser-defined functionalized graphene photodetectors

**DOI:** 10.1126/sciadv.1602617

**Published:** 2017-05-26

**Authors:** Adolfo De Sanctis, Gareth F. Jones, Dominique J. Wehenkel, Francisco Bezares, Frank H. L. Koppens, Monica F. Craciun, Saverio Russo

**Affiliations:** 1 Centre for Graphene Science, College of Engineering, Mathematics and Physical Sciences, University of Exeter, EX4 4QL Exeter, UK; 2 Institut de Ciències Fotòniques (ICFO), Mediterranean Technology Park, 08860 Castelldefels, Barcelona, Spain.

**Keywords:** graphene, photodetector, LDR, functionalization, SNOM, photovoltaic, hot-carriers, RAMAN, sensing

## Abstract

Graphene-based photodetectors have demonstrated mechanical flexibility, large operating bandwidth, and broadband spectral response. However, their linear dynamic range (LDR) is limited by graphene’s intrinsic hot-carrier dynamics, which causes deviation from a linear photoresponse at low incident powers. At the same time, multiplication of hot carriers causes the photoactive region to be smeared over distances of a few micrometers, limiting the use of graphene in high-resolution applications. We present a novel method for engineering photoactive junctions in FeCl_3_-intercalated graphene using laser irradiation. Photocurrent measured at these planar junctions shows an extraordinary linear response with an LDR value at least 4500 times larger than that of other graphene devices (44 dB) while maintaining high stability against environmental contamination without the need for encapsulation. The observed photoresponse is purely photovoltaic, demonstrating complete quenching of hot-carrier effects. These results pave the way toward the design of ultrathin photodetectors with unprecedented LDR for high-definition imaging and sensing.

## Teaser

Quenching thermoelectric effects in graphene leads to an extraordinary increase of the linear dynamic range in photodetectors.

## INTRODUCTION

Intense research activity on graphene-based photodetectors (*1*) has demonstrated a unique range of properties, including mechanical flexibility (*2*), large operating bandwidth (*3*), and broadband spectral response. However, state-of-the-art inorganic (Si, Ga, GaAs, etc.) photodetectors currently exhibit a linear response over a larger range of optical powers, as compared to graphene. This is due to the comparatively small density of states in graphene at energies below 1 eV. Furthermore, the thermal diffusion of photogenerated carriers has been found to dominate photocurrent signals measured by graphene-based photodetectors (*4*–*6*). These strong photothermoelectric (PTE) effects enable multiplication of hot carriers but also cause photoresponsive regions to be smeared out over distances exceeding 2 μm (*5*–*7*). The narrow linear dynamic range (LDR) and the size of the photoresponsive regions in graphene photodetectors limit integration of graphene pixels in high-resolution sensing and video imaging applications.

Chemical functionalization (*8*) is a largely unexplored route to overcoming the intrinsic limitations on sensing introduced by hot-carrier dynamics in pristine graphene, where the limited size of the Fermi surface imposes tight constraints to the carrier relaxation dynamics (*9*). Although attempts have been made to use chemical functionalization to engineer p-n junctions in graphene (*10*, *11*) and selectively define photoresponsive regions (*2*, *12*, *13*), no major improvements have been shown compared to pristine graphene devices; therefore, several challenges remain. These include finding forms of functionalization that give ultrahigh values of charge doping and are also air-stable. Functionalization of graphene with FeCl_3_ has been found to result in record-high levels of hole-doping (≈ 1 × 10^15^ cm^−2^) with a room temperature electrical conductivity up to 1000 times larger than that of pristine graphene while maintaining equivalent absorption over the visible wavelength range (*14*, *15*). At the same time, an unforeseen stability under harsh environmental conditions (*16*), the ease of large-area processing (*15*), and the promise for efficient coupling of telecommunication wavelength light to electrical signals through surface plasmons make this material uniquely suited for exploring novel optoelectronic applications. The development of a new generation of imaging arrays with unprecedented LDR and pixel density, which do not use any thermal isolation or electrostatic gating at high voltages and are stable under both ambient and harsh conditions, would bring imaging and sensing technologies to new frontiers.

Here, we demonstrate micrometer- and nanometer-scale planar photoresponsive junctions, which are directly written in the host material using focused laser light. Characterization of photocurrent signals reveals a purely photovoltaic (PV) response and an LDR as large as 44 dB, which is at least 4500 times larger than that of any previously reported graphene photodetector (*3*, *9*, *17*–*20*). Crucially, these detectors exhibit marked stability under atmospheric conditions without any form of encapsulation and maintain a broad spectral response from ultraviolet A (UVA) to midinfrared (MIR) wavelengths. Using emerging nanophotonics tools, such as near-field photocurrent nanoscopy, we can surpass the diffraction-limited resolution of far-field methods and define photoresponsive junctions smaller than half the laser wavelength used.

The light-assisted design of integrated and atomically thin optoelectronic circuits is a step forward to a new frontier in high-definition sensing applications, whereas FeCl_3_-intercalated few-layer graphene (FeCl_3_-FLG) defines a new paradigm in ultrathin, high-LDR photodetectors.

## RESULTS AND DISCUSSION

### Preparation of laser-defined junctions

**Fig. 1:**
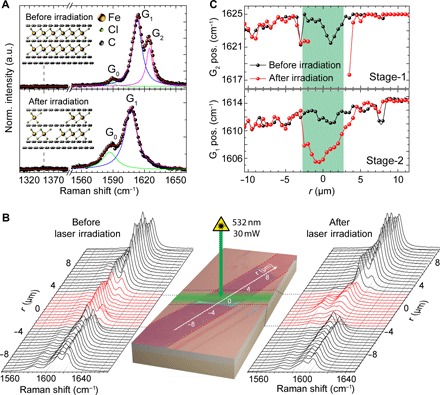
Raman spectroscopy study of structural changes in laser-irradiated FeCl_3_-FLG. (**A**) G bands in FeCl_3_-FLG before (top) and after (bottom) exposure to a 30-mW laser for 3 s (λ = 532 nm). Experimental data (black dots) is shown alongside a superposition of Lorentzian fits to the G_0_, G_1_, and G_2_ peaks (red line). (**B**) Optical micrograph of the FeCl_3_-FLG flake (red dotted lines) with the laser-irradiated region highlighted (green). Raman spectra are acquired along *r* before (left) and after (right) FeCl_3_ displacement. (**C**) G_1_ (bottom) and G_2_ (top) peak positions representing stages 1 and 2 intercalated states, respectively. Data points are Lorentzian fits of the spectral peaks in (B).

The starting material to achieve our goal is an intercalated four-layer graphene flake with FeCl_3_ introduced only between the top three carbon layers. Intercalation of FeCl_3_ molecules into mechanically exfoliated FLG on a Si/SiO_2_ substrate was conducted using a previously reported method (*14*) in a two-zone furnace (see Materials and Methods). A typical Raman spectrum of this system shows the G_0_ peak at 1580 cm^−1^ due to the E_2g_ phonon mode of pristine graphene as well as the blue-shifted G_1_ = 1615 cm^−1^ and G_2_ = 1625 cm^−1^ peaks of the same mode caused by the charge doping of FeCl_3_ molecules adjacent to only one side of a graphene layer (stage 2) or sandwiching the carbon atoms (stage 1) (see Fig. 1A). Upon its exposure to 532-nm laser light with an incident power of 15.3 MW/cm^2^ for 3 s, we observe drastic modifications in the Raman G band: a pronounced downshift of the G peak positions; a reduction of their full width at half maximum; the disappearance of the G_2_ peak; and the emergence of the G_0_ peak (see Fig. 1A). All these changes indicate a reduction in hole doping caused by laser-induced displacement of FeCl_3_, with the disappearance of the G_2_ peak stemming from the complete removal of stage 1 intercalation. Finally, the absence of a defect-related Raman peak demonstrates that this functionalization can truly sustain laser powers more than 300 times higher than those of pristine graphene (section S1).

To ascertain the effectiveness of laser irradiation as a method for locally tailoring FeCl_3_ intercalation in graphene, we exposed a 5.5-μm-wide section of the intercalated flake to a raster laser scan (15.3 MW/cm^2^ for 3 s in 0.5-μm steps). Raman spectra were collected at incrementally spaced locations across the laser-exposed region both before and after illumination, as shown in Fig. 1B. Comparing the spectral profiles at each location, it is apparent that all irradiated regions undergo a substantial degree of deintercalation. In Fig. 1C, we quantify changes in chemical structure across the entire laser-exposed region by analyzing the positions of the G_1_ and G_2_ peaks along a 21-μm line scan. Uniform removal of the G_2_ peak from the entire rastered region demonstrates that FeCl_3_ molecules may be displaced from arbitrarily mapped areas. The degree of intercalation remains unchanged away from the irradiated area, with the resolution of FeCl_3_ displacement defined by the laser spot profile. The marked effectiveness of laser-induced deintercalation over a significant fraction of the FeCl_3_-FLG flake area presents an elegant method, akin to optical lithography, which can be used to locally customize the chemical functionalization of graphene layers.

**Fig. 2:**
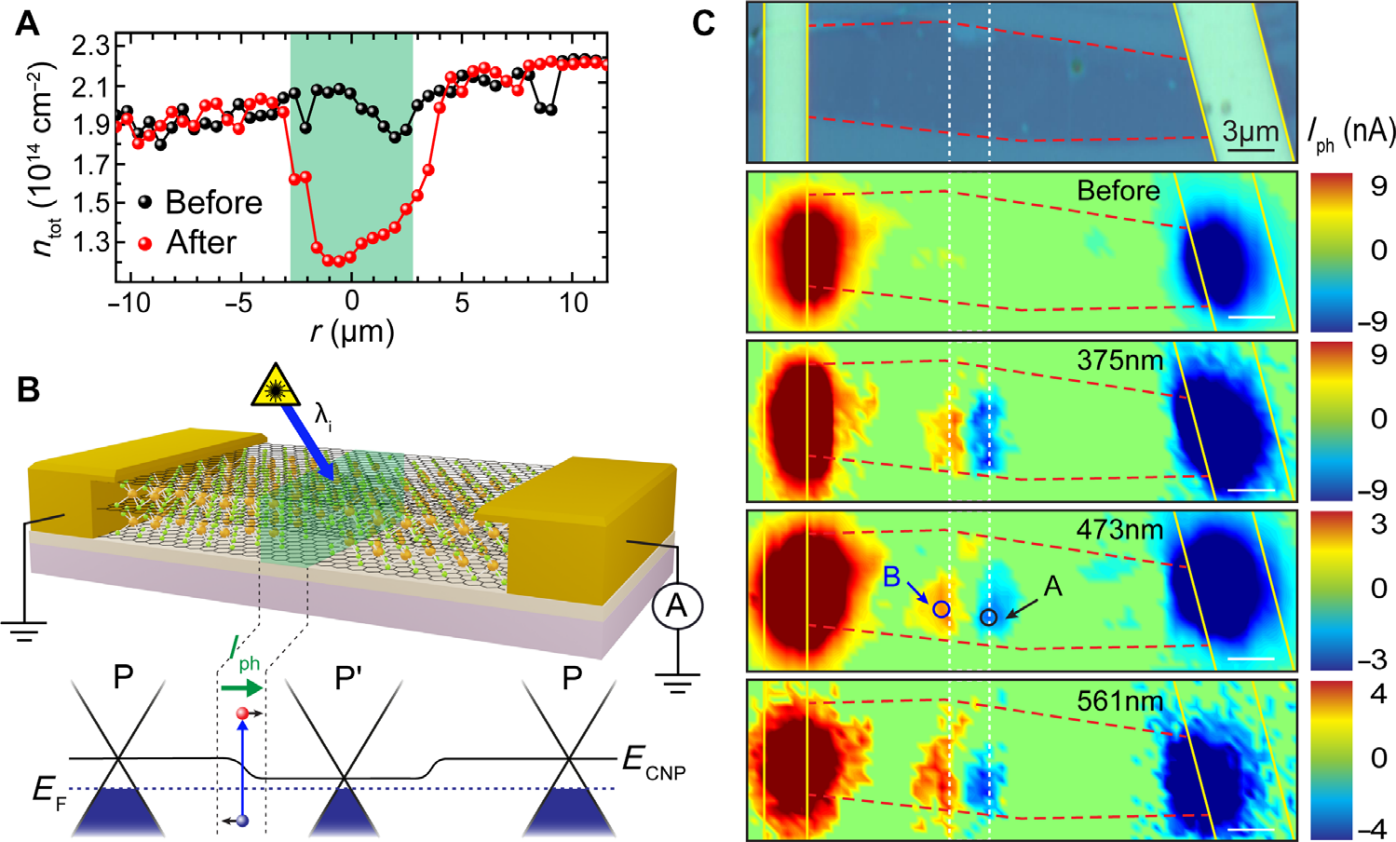
Scanning photocurrent microscopy of p-p′ junctions in FeCl_3_-FLG. (**A**) Total charge carrier concentration before and after laser-assisted displacement of FeCl_3_, estimated from G peak positions in Fig. 1C. (**B**) Short-circuit configuration (top) for scanning photocurrent measurements of a p-p′-p junction (p-p′ region in green). Schematic band structure (bottom) of each region shows photogenerated carriers drifting under a chemical potential gradient. (**C**) Optical micrograph (top) of an FeCl_3_-FLG flake (red dashed lines) with Au contacts (yellow lines). Scanning photocurrent maps (bottom) before and after selective laser-assisted displacement of FeCl_3_ (white dashed lines). The photoresponse is measured for excitation wavelengths of 375, 473, and 561 nm.

The shift of the Raman G peak is quantitatively translated into a charge density using the model developed by Lazzeri *et al*. (*21*) and Das *et al*. (*22*) with an accuracy of ±10%, as shown by independent characterization of charge density from quantum oscillations in magnetoconductance (*14*, *15*). We find that the laser irradiation of FeCl_3_ causes a reduction in charge density of up to Δ*p*_tot_ ≈ − 0.6 × 10^14^ cm^−2^ (Fig. 2A), which agrees with electrical measurements showing a 170% increase in resistivity over the modified area (see section S4). Hence, the abrupt change in hole concentration at the boundaries of the laser-exposed region defines sharp p-p′ junctions (see section S2.5 for data on additional devices).

### Optoelectronic response of laser-defined p-p′ junctions

Inspired by the rich variety of charge transfer processes, which has enabled a revolution in semiconductor heterostructure applications, we examined the optoelectronic response of these laser-defined junctions in FeCl_3_-FLG. Laser light focused to a beam spot diameter of 1.0 μm at 300 μW was rastered over the device surface while measuring photocurrent signals (see Fig. 2B). Photocurrent maps are provided in Fig. 2C for a variety of excitation wavelengths. The sign convention of the photocurrent has been carefully configured so that a positive signal indicates the drift of holes from the left to the right electrode (section S5.4). As expected, for uniform doping, no significant photocurrent is observed in FeCl_3_-FLG before laser patterning. However, when a p-p′-p junction is defined by laser-assisted displacement of FeCl_3_, a photocurrent as large as 9 nA is measured at each of the lateral interfaces.

A multitude of physical mechanisms can give rise to a photoresponse. Of these mechanisms, two play a major role in graphene-based photodetectors. They are the PTE and the PV effects (*1*). The PTE effect originates from a difference in Seebeck coefficients, Δ*S* = (*S*′ − *S*), across a graphene junction formed by regions with a differing density of states. If the junction is illuminated, a local increase of temperature (Δ*T*) results in the diffusion of carriers and an opposing photovoltage (*V*_PTE_ = Δ*S* Δ*T*) is generated. Hot-carrier dynamics are generally recognized to dominate photocurrent generation in supported graphene devices because of inefficient cooling of electrons with the lattice (*5*, *6*). For the PV effect, incident photons generate a density (*n*_ph_) of carriers, which, in the presence of an in-built electric field, are separated and induce current at the electrodes (Fig. 2B). Other mechanisms, such as the bolometric, photogating, and Dyakonov-Shur effects require an externally applied voltage (*1*) and are therefore not active in the short circuit configuration of our measurements (Fig. 2B).

**Fig. 3:**
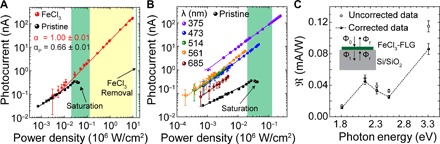
Characterization of photocurrent at p-p′ junctions in FeCl_3_-FLG. (**A**) Photocurrent produced by λ = 473 nm excitation as a function of incident power density measured at a laser-defined p-p′ junction and for pristine monolayer graphene (black). Power-law exponents (*I*_ph_ ∝ *P*^α^) are detailed for each data set with fits shown as solid lines. Powers within the range at which photocurrent in pristine graphene that has been reported to saturate are highlighted in green (see table S1). Yellow-shaded area represents the extended range of FeCl_3_-FLG. (**B**) Photocurrent measured at the p-p′ junction A in Fig. 2B using various excitation wavelengths. Solid lines are linear fits (see main text). (**C**) Spectral responsivity of a p-p′ junction in FeCl_3_-FLG shown with (filled circles) and without (open circles) correction for reflections from the Si/SiO_2_ substrate (section S6) are extrapolated from (B). The dashed line is a guide to the eye. Inset: Schematic of the model used to correct ℜ(λ) for substrate reflections. Power density and responsivity values are calculated considering the area illuminated by the laser spot (see Materials and Methods).

A first insight into the microscopic mechanism behind the observed photocurrent can be gained by comparing the laser power dependence in pristine and intercalated graphene. Figure 3A shows a typical power dependence for the photocurrent (*I*_PH_ ∝ *P*^α^) generated in one of several measured monolayer graphene devices (section S2.4), where α = 2/3 was obtained with 10 mV applied between the source and drain. On the other hand, the photoresponse of FeCl_3_-FLG is strikingly different from that of pristine graphene, exhibiting a linear dependence extending beyond three logarithmic decades of incident laser power. The observed difference originates from the charge carrier dynamics. More specifically, in pristine graphene, the chemical potential (μ) lies close to the charge neutrality point, and the small Fermi surface imposes tight constraints on the maximum energy lost through momentum-conserving acoustic phonon emission (Δ*E*_ac_ < 2*ℏv*_s_*k*, where *v*_s_ ~ 2 × 10^4^ ms^−1^ is the acoustic phonon speed and *k* is the hot carrier wave number) (*23*). As a result, photoexcited carriers reach a steady-state temperature far above that of the lattice (*T*_h_ >> *T*_l_) and are instead cooled via short-range “supercollision” processes at sites of disorder (*9*, *24*). If the PTE effect is similarly responsible for photocurrent in FeCl_3_-FLG, the steady-state temperature of hot carriers must lie significantly closer to that of the lattice (*T*_h_ − *T*_l_ << *T*_l_) to justify the observed linear power dependence (*9*). A reduction in *T*_h_ can be explained by the ultrahigh levels of charge density (up to 3 × 10^14^ cm^−2^ per layer) achieved through FeCl_3_ intercalation (*14*); the expanded Fermi surface enhances Δ*E*_ac_ to as much as 60 times that of pristine graphene, accelerating the cooling of photogenerated charges. On the other hand, the small temperature gradients present at these highly doped junctions could diminish thermoelectric currents so much that they become negligible compared to signals generated by the PV effect. A linear power dependence would also be expected in this case (*25*), provided that the incident light intensity is sufficiently low so as to not affect the average lifetime (τ) of photogenerated carriers. The observation of photocurrent with a linear dependence on incident power therefore indicates enhanced cooling of hot carriers in FeCl_3_-FLG but, as other studies have suggested (*19*), cannot be used independently to distinguish between PTE and PV effects.

### Photovoltaic effect in FeCl_3_-FLG junctions

To identify the origin of photocurrent at p-p′ junctions of FeCl_3_-FLG, we adapt the model of Song *et al*. (*5*) to calculate the relative contributions of the PTE and PV effects (section S5). The photocurrent produced in a p-p′ junction located in the middle of an FeCl_3_-FLG channel (length *L* and width *W*) illuminated by a laser (spot diameter *l*_0_) is

$$I_{\text{ph}}=\int_{0}^{W}\int_{-\frac{L}{2}}^{\frac{L}{2}}[S_{\left(x,y\right)}\nabla T_{\left(x,y\right)}-\sigma_{\left(x,y\right)}^{-1}n_{\text{ph}(x,y)}\eta \nabla \mu_{\left(x,y\right)}]\frac{dydx}{RW}$$ (1)

where *R* is the resistance of the graphene layer and *η* is the carrier mobility. For a doped graphene layer with a charge carrier density above *n* ≈ 3 × 10^13^ cm^−2^, the Bloch-Grüneisen temperature (*T*_BG_ = Δ*E*_ac_/*k*_B_) exceeds 300 K (*26*). Therefore, under continuous wave illumination, where Δ*T* is typically just a few kelvin (*7*), bottlenecks in electron-acoustic phonon coupling are alleviated in FeCl_3_-FLG. The increased efficiency of momentum-conserving acoustic phonon emission renders supercollisions irrelevant to hot-carrier cooling processes and reduces the average cooling length (ζ) from several micrometers (*5*, *7*) to approximately 200 nm. Hence, for a typical device, ζ << *L*/2. Using the low-energy density of states for monolayer graphene and a minimum conductivity of σ_min_ ≈ 4*e*^2^/*h* (*27*), we express the conductivity of each decoupled layer as a function of its chemical potential σ(μ) = σ_min_(1 + μ^2^/Λ^2^), where Λ ≈ 140 meV. The Mott relation for thermopower (*27*) and a solution to the heat equation, which assumes nondivergent current densities, (*5*) are then used with Eq. 1 to estimate the relative magnitudes of PTE and PV currents from the electrical properties on either side of the p-p′ junction

$$\frac{I_{\text{PTE}}}{I_{\text{PV}}}=\frac{2ek_{B}T_{h}l_{0}\sigma_{\text{min}}}{\mu \mu^{\prime }\eta \tau \Lambda }\cdot \frac{\left[\mu^{\prime }(1-\frac{\sigma_{\text{min}}}{\sigma })-\mu (1-\frac{\sigma_{\text{min}}}{\sigma^{\prime }})\right]}{\left(\frac{\sigma }{\zeta }+\frac{\sigma^{\prime }}{\zeta^{\prime }})\cdot [{\text{tan}}^{-1}(\frac{\mu }{\Lambda })-{\text{tan}}^{-1}(\frac{\mu^{\prime }}{\Lambda })\right]}$$ (2)

with 1 ps < τ < 2 ps, which is in agreement with pump-probe spectroscopy measurements (*28*) (see section S5), and all material parameters are averaged over the device width. For each of the decoupled monolayers in the four-layer flake, where the most prominent changes in chemical potential occur after laser writing, we calculate *I*_PTE_/*I*_PVE_ ≈ − 0.06 using Eq. 2. Thermalization of hot carriers therefore makes a negligible contribution to the total photocurrent generated at FeCl_3_-FLG p-p′ junctions and acts in the opposite direction to dominant photovoltaic processes. Opposing photocurrents at p-p′ junctions have previously been predicted in monolayer graphene transistors with split electrostatic gates (*5*) and can be understood intuitively by considering that the movement of photogenerated charge carriers is governed by local gradients in chemical potential for photovoltaic currents and by local gradients in Seebeck coefficient for thermoelectric currents. Following the Mott relation (*S* ∝ −σ^−1^ (*d*σ/dμ)), the density of states of graphene dictates that these gradients will always point in opposite directions so long as the chemical potentials on each side of a photoactive junction are both situated in the valence band (p-p′ junctions) or in the conduction band (n-n′ junctions) away from the charge neutrality point. As a result of these findings, we take the direction of photocurrent signals shown in Fig. 2C (where carriers drift according to the local potential gradient at p-p′ interfaces) as direct evidence of a purely photovoltaic response in laser-written FeCl_3_-FLG detectors.

Through chemical functionalization, the selective quenching of thermoelectric processes in graphene could prove to be a highly useful tool for extending the use of graphene-based light sensors beyond microbolometers and modulators suitable for infrared wavelengths. Pixels of FeCl_3_-FLG–based photodetectors would not require thermal isolation and could be packed to a far higher density than undoped graphene monolayers, making them well-suited for imaging applications over a broad spectral range.

### Extraordinary LDR

The purely PV response in FeCl_3_-FLG detectors is characterized by an extraordinary LDR. The noise-equivalent power (NEP) of our device was measured to be 4 kW/cm^2^ (see section S2.2), thus resulting in an LDR of 44 dB. This is 4500 times larger than that of previously reported graphene photodetectors (LDR ≈ 7.5 dB) (*3*) and ~800 times larger than that of other functionalized graphene devices (LDR ≈ 15 dB) (*13*). In table S1, we show a comparison of the maximum saturation power and LDR for different devices reported in literature (see also section S2.3 for a comparative study of detectors).

To further assess the suitability of FeCl_3_-FLG for optoelectronic applications, we have characterized the photoresponse at these p-p′ junctions over a wide range of light intensities and wavelengths. Figure 3B shows the power dependence of photocurrent measured at a p-p′ junction in FeCl_3_-FLG for various wavelengths of incident light ranging from UVA (375 nm) to red (685 nm). Fits of the power exponent at each wavelength give the following: α_375_ = 0.99 ± 0.01, α_473_ = 1.05 ± 0.06, α_514_ = 0.97 ± 0.03, α_561_ = 0.99 ± 0.01, and α_685_ = 0.95 ± 0.05. For the multitude of FeCl_3_-FLG devices measured, we observed no deviation from a strictly linear power dependence in the whole measured power range. This indicates that the ultrahigh degree of charge carrier doping introduced by FeCl_3_ intercalation acts as a unique stable method for quenching thermoelectric effects and fixing the photoresponse to an extended linear dynamic regime, avoiding the sensitivity to processing methods and environmental conditions, which pristine graphene photodetectors (*3*, *9*) inevitably experience. In Fig. 3C, the spectral responsivity, ℜ(λ) = *I*_ph_/*P*_opt_(λ), of a p-p′ junction is displayed with and without correcting for reflections from the Si/SiO_2_ substrate (section S6). The photoresponse remains markedly consistent across the entire visible range, where ℜ(λ) varies by only one order of magnitude, with values >0.1 mA/W, which are typical for high-end all-graphene photodetectors (*1*). Of particular interest is the increase in responsivity toward UVA wavelengths, a region where the performance of silicon photodiodes decreases. We attribute the extended LDR to accelerated carrier cooling and the enhanced responsivity to an increased high-energy density of states introduced by FeCl_3_ intercalation of graphene (*28*). This consistent proportionality between output electrical signal and incident optical power over a broad spectral range makes FeCl_3_-FLG–based photodetectors ideally suited to radiometry and spectroscopy applications.

### Below the diffraction limit

**Fig. 4:**
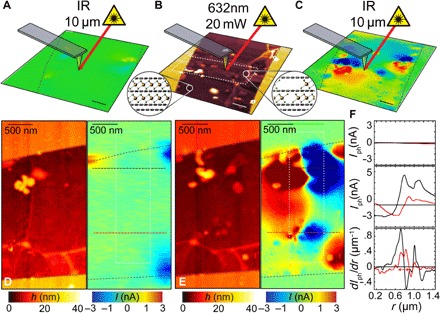
High-resolution photoactive junctions in FeCl_3_-FLG defined using near-field scanning microscopy. (**A**) Spatial map of photocurrent in a uniformly doped graphene flake before laser-assisted deintercalation. (**B**) AFM topography and (**C**) scanning photocurrent maps of the FeCl_3_-FLG flake after laser-assisted deintercalation by a λ = 632 nm laser scanned over a 500-nm long region (white dashed lines). Insets: Illustrations of the chemical structure in p- and p′-doped regions. Schematic of the excitation wavelength focused on a metallized AFM tip in each measurement are included in (A) to (C); outlines of the flake are superimposed (black dashed lines). Scale bars, 500 nm. Magnified concurrent AFM topography and scanning photocurrent maps are shown before (**D**) and after (**E**) laser writing. (**F**) Line scans of photocurrent measured across laser-defined p-p′-p junctions [(D) and (E), red and black dashed lines] before (top) and after (middle) displacement of molecules. First-derivative plots of the photocurrent signal after displacement (bottom) show a peak-to-peak distance of 250 nm between adjacent p-p′ junctions (red arrows). All photocurrent measurements were taken in short-circuit configuration.

The spatial resolution of FeCl_3_ displacement at the engineered p-p′ junctions is determined by the profile of the laser spot used for patterning. In far-field optical microscopy, spot sizes are dictated by the Abbe diffraction limit [~λ/(2NA), where NA is the numerical aperture of the objective]. To explore the density to which graphene-based imaging pixels may be packed in the absence of hot-carrier effects, we use scattering-type near-field optical microscopy (s-SNOM; see Materials and Methods) to define photoactive junctions below the Abbe limit. This technique has been used extensively to study the plasmonic (*29*) and optoelectronic (*30*) response of graphene-based devices. Figure 4 (A to C) shows photocurrent maps using a λ = 10 μm excitation source taken before and after FeCl_3_ displacement by a λ = 632 nm laser. Planar junctions exhibiting a photovoltaic response are readily defined with a peak-to-peak separation of just 250 nm (Fig. 4F), while concurrent topography mapping (Fig. 4, D and E) indicates that the flake surface remains undamaged. Furthermore, the photocurrent is stronger near the edges of the flake, suggesting that the deintercalation process is due to the displacement of FeCl_3_ molecules in the plane of graphene that are removed from the edges. The absorption of photons with energy *E* << 2μ in FeCl_3_-FLG highlights the role of transitions to the π band from localized states introduced by FeCl_3_, as predicted by density functional theory calculations (*31*). This prevents Pauli blocking of long wavelengths and maintains a broadband spectral response, up to MIR wavelengths, in these novel photodetectors.

## CONCLUSIONS

In conclusion, laser patterning is an elegant method for creating photoresponsive junctions in intercalated few-layer graphene. Photoresponsive junctions in FeCl_3_-FLG are engineered on the submicrometer scale and remain highly stable under atmospheric conditions and intense light exposure. This presents a unique opportunity relative to other methods of chemical functionalization, whereby photocurrent mechanisms are reliably pinned to produce a linear response over broad ranges of power and wavelength with no requirement for encapsulation from the environment. These junctions show an extraordinary LDR up to 44 dB, more than 4500 times larger than that of other graphene photodetectors, which can operate at incident optical powers up to 10^4^ kW/cm^2^ in the whole visible range, in the near-UV, and at MIR wavelengths. Further enhancements in responsivity can be achieved through the use of an increased number of intercalated graphene layers and optimization of the deintercalation process to maximize the chemical potential gradient at p-p′ junctions. Uniform intercalation of FeCl_3_ throughout large-area graphene films of a uniform layer number will be crucial for implementing these findings in practical applications. To this end, intercalation of large-area chemical vapor deposition (CVD)–grown graphene has already been demonstrated (*15*, *32*, *33*), and roll-to-roll processing of graphene (*34*) is readily applicable to intercalated films. Compact pixel arrays could be realized using vertical circuitry equivalent to buried channels in complementary metal-oxide semiconductor technology, where vias connect between pixels on the substrate surface and laterally running interconnects dispersed over several buried levels. These findings provide exciting prospects for light detection in laser-induced plasmas, UV photocatalytic water sanitation processes, and high-precision manufacturing. In these environments, these novel sensors could eliminate the need for attenuating optics in the detection of ultrabright light signals with high spatial resolution.

## MATERIALS AND METHODS

### Device fabrication

Few-layer graphene flakes were mechanically exfoliated from natural graphite on a p-doped silicon substrate with a 280-nm surface oxide. Intercalation with FeCl_3_ was conducted in a two-zone furnace using a previously demonstrated vapor transport method (*14*). Electrical contacts to the flakes were defined by standard electron-beam lithography, thermal deposition of Cr/Au (5/50 nm), and lift-off in acetone.

### Raman spectroscopy

Raman spectroscopy measurements used to characterize the degree of intercalation in FeCl_3_-FLG were performed in atmosphere and at room temperature (see the Supplementary Materials). Raman spectra were acquired with a Renishaw spectrometer equipped with a 532-nm laser focused to a 1.0-μm spot through a 50× objective lens. An incident power of 1 mW was used for all measurements, and spectra were recorded with a 2400 g/mm grating. A charge-coupled device acquisition time of 5 s was used.

### Photocurrent measurements

A continuous-wave laser beam from a multiwavelength (Coherent OBIS 375LX, 473LS, 514LX, 561LS and Omicron LuxX 685) solid-state laser diode array was focused onto the sample through a 50× lens, producing a spot size of 1.0 μm. A high-resolution microscope stage (minimum step size of 0.05 μm) was used to produce spatial maps of the photocurrent. Electrical measurements were performed in short-circuit (zero bias) configuration using a DL Instruments Model 1211 current amplifier connected to a Signal Recovery model 7124 digital signal processing lock-in amplifier. The lasers were modulated at a frequency of 73.3 Hz with a transistor-transistor logic signal from a direct digital synthesizer function generator, which was used as a reference signal for the lock-in. All measurements were performed under ambient conditions (*T* = 300 K, *P* = 1 atm) in air. The laser power was varied from 1.5 μW to 1 mW by analog modulation of the laser diodes and the use of neutral density filters along the beam path. All the devices studied have been measured in air over a time scale longer than 1 year, during which no change in the photoresponse was observed.

### LDR calculation

The LDR is defined as

$$\text{LDR}=10\times {\text{log}}_{10}(\frac{P_{\text{sat}}}{\text{NEP}})[\text{dB}]$$ (3)

where the NEP is defined as the power at which the signal-to-noise ratio has a value of 1. The NEP can be measured directly or computed as $\text{NEP}=S_{I}/ℜ[W/\sqrt{\text{Hz}}]$, where *S*_*I*_ is the root mean square current noise (in $A/\sqrt{\text{Hz}}$), and ℜ is the responsivity of the photodetector (in A/W).

### s-SNOM measurements

s-SNOM involves focusing a laser onto a metallized atomic force microscope (AFM) tip, which creates a strong, exponentially decaying field at its apex. The tip is then scanned across the sample, operating in tapping mode, allowing parameters, including topography and scattered light emission, to be measured with subwavelength resolution (*35*–*37*). If the device is contacted as in this work, the local photocurrent, produced by the light focused at the tip, could be measured with the same resolution. s-SNOM measurements were performed using a commercially available system from Neaspec GmbH. The AFM tips used were commercially available metal-coated tips with average radii of 25 nm. Our system was equipped with a tunable CO_2_ laser and a visible wavelength HeNe laser. Here, the CO_2_ laser was used to probe the optical near-field signal of our samples, whereas the visible laser was used only for laser patterning of the p-p′ junctions in our devices. Concurrent photocurrent and AFM topography measurements were performed in short-circuit configuration using the CO_2_ laser before and after laser patterning.

## Supplementary Material

http://advances.sciencemag.org/cgi/content/full/3/5/e1602617/DC1

## SUPPLEMENTARY MATERIALS

Supplementary material for this article is available at http://advances.sciencemag.org/cgi/content/full/3/5/e1602617/DC1 section S1. Supplementary data on laser irradiation section S2. Supplementary photocurrent measurements section S3. Power dependence of the photothermoelectric and photovoltaic effects section S4. Estimation of chemical potential and conductivity for decoupled graphene layers section S5. Physical explanation for a purely photovoltaic response section S6. Correction of responsivity spectra for substrate reflections fig. S1. Inferred stacking order of four-layer FeCl_3_-FLG. fig. S2. Calibration of laser-induced displacement of FeCl_3_. fig. S3. Bandwidth of a laser-written FeCl_3_-FLG junction device. fig. S4. NEP of laser-written FeCl_3_-FLG junction device. fig. S5. Characterization of supported pristine graphene devices. fig. S6. Additional measurements of photocurrent in supported pristine graphene devices. fig. S7. Photoresponse at p-p′ junction in FLG. fig. S8. Calculation of the carrier concentration and chemical potential at p-p′ interfaces of FeCl_3_-FLG. fig. S9. Direction of photocurrent at p-p′ junctions of FeCl_3_-FLG. fig. S10. Correction of spectral responsivity for substrate reflections. table S1. LDR of graphene and functionalized graphene devices. table S2. Summary of power-law exponents possible for photocurrent originating from the photothermoelectric effect. table S3. Corrections to responsivity for the laser wavelengths used in this work. References (*38*–*47*)
