# Supplementary material for: Extraordinary linear dynamic range in laser-defined functionalized graphene photodetectors
Source: Sci Adv. 2017 May 26;3(5):e1602617. doi: 10.1126/sciadv.1602617 (PMC5446211; doi:10.1126/sciadv.1602617)
Supplement: http://advances.sciencemag.org/cgi/content/full/3/5/e1602617/DC1 [file supp_3_5_e1602617__index.html]

Science Advances | Science Advances

## Supplementary Materials

**This PDF file includes:**

- section S1. Supplementary data on laser irradiation
- section S2. Supplementary photocurrent measurements
- section S3. Power dependence of the photothermoelectric and photovoltaic effects
- section S4. Estimation of chemical potential and conductivity for decoupled graphene layers
- section S5. Physical explanation for a purely photovoltaic response
- section S6. Correction of responsivity spectra for substrate reflections
- fig. S1. Inferred stacking order of four-layer FeCl3-FLG.
- fig. S2. Calibration of laser-induced displacement of FeCl3.
- fig. S3. Bandwidth of a laser-written FeCl3-FLG junction device.
- fig. S4. NEP of laser-written FeCl3-FLG junction device.
- fig. S5. Characterization of supported pristine graphene devices.
- fig. S6. Additional measurements of photocurrent in supported pristine graphene devices.
- fig. S7. Photoresponse at p-p′ junction in FLG.
- fig. S8. Calculation of the carrier concentration and chemical potential at p-p′ interfaces of FeCl3-FLG.
- fig. S9. Direction of photocurrent at p-p′ junctions of FeCl3-FLG.
- fig. S10. Correction of spectral responsivity for substrate reflections.
- table S1. LDR of graphene and functionalized graphene devices.
- table S2. Summary of power-law exponents possible for photocurrent originating from the photothermoelectric effect.
- table S3. Corrections to responsivity for the laser wavelengths used in this work.
- References (*38–47*)

Download PDF

**Files in this Data Supplement:**

- Adobe PDF - 1602617\_SM.pdf
