# Supplementary material for: Extraordinary linear dynamic range in laser-defined functionalized graphene photodetectors
Source: Sci Adv. 2017 May 26;3(5):e1602617. doi: 10.1126/sciadv.1602617 (PMC5446211; doi:10.1126/sciadv.1602617)
Supplement: http://advances.sciencemag.org/cgi/content/full/3/5/e1602617/DC1 [file 1602617_SM.pdf]

Science Advances

AAAS

advances.sciencemag.org/cgi/content/full/3/5/e1602617/DC1

Supplementary Materials for

Extraordinary linear dynamic range in laser-defined functionalized graphene photodetectors

Adolfo De Sanctis, Gareth F. Jones, Dominique J. Wehenkel, Francisco Bezares, Frank H. L. Koppens, Monica F. Craciun, Saverio Russo

Published 26 May 2017, *Sci. Adv.* **3**, e1602617 (2017)
DOI: 10.1126/sciadv.1602617

This PDF file includes:

- section S1. Supplementary data on laser irradiation
- section S2. Supplementary photocurrent measurements
- section S3. Power dependence of the photothermoelectric and photovoltaic effects
- section S4. Estimation of chemical potential and conductivity for decoupled graphene layers
- section S5. Physical explanation for a purely photovoltaic response
- section S6. Correction of responsivity spectra for substrate reflections
- fig. S1. Inferred stacking order of four-layer $FeCl_3$-FLG.
- fig. S2. Calibration of laser-induced displacement of $FeCl_3$.
- fig. S3. Bandwidth of a laser-written $FeCl_3$-FLG junction device.
- fig. S4. NEP of laser-written $FeCl_3$-FLG junction device.
- fig. S5. Characterization of supported pristine graphene devices.
- fig. S6. Additional measurements of photocurrent in supported pristine graphene devices.
- fig. S7. Photoresponse at p-p′ junction in FLG.
- fig. S8. Calculation of the carrier concentration and chemical potential at p-p′ interfaces of $FeCl_3$-FLG.
- fig. S9. Direction of photocurrent at p-p′ junctions of $FeCl_3$-FLG.
- fig. S10. Correction of spectral responsivity for substrate reflections.
- table S1. LDR of graphene and functionalized graphene devices.
- table S2. Summary of power-law exponents possible for photocurrent originating from the photothermoelectric effect.
- table S3. Corrections to responsivity for the laser wavelengths used in this work.
- References (*38*–*47*)

section S1. Supplementary data on laser irradiation

S1.1 Determination of the stacking order in $FeCl_3$-FLG

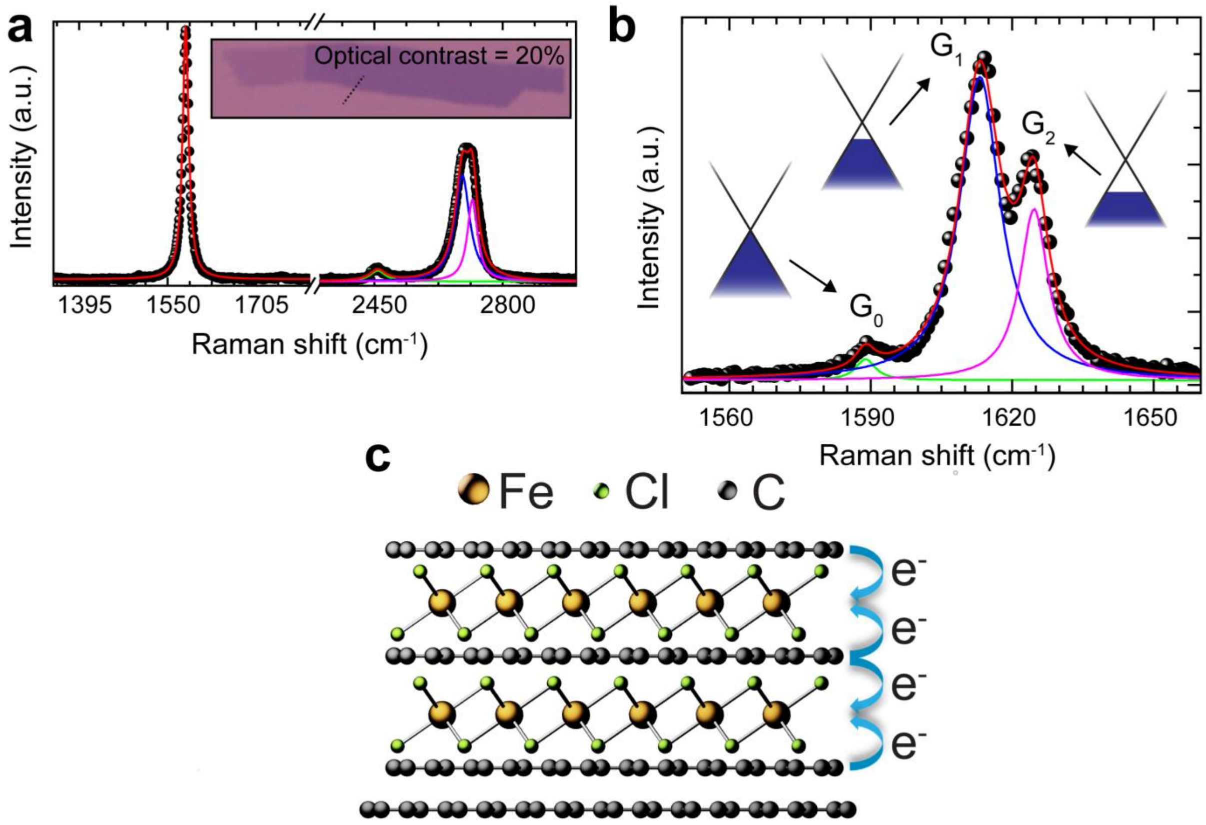

fig. S1. Inferred stacking order of four-layer $FeCl_3$-FLG. (**a**) Raman spectrum of the same four-layer graphene flake before intercalation with $FeCl_3$. Inset, Image analysis of an optical micrograph shows a 20% contrast between the flake and Si/SiO_2 before intercalation. (**b**) Raman spectrum acquired after $FeCl_3$ intercalation, the levels of p-doping corresponding to the G_0, G_1 and G_2 peaks are illustrated. (**c**) Stacking order of the $FeCl_3$-FLG flake presented in Figs. 1 to 3 (main text).

Using a combination of optical microscopy and Raman spectroscopy it is possible to determine the stacking order of the $FeCl_3$-FLG. We consider the specific case of the flake discussed in the main text in Fig. 1A. This is a four-layer graphene as inferred from the optical contrast relative to the Si/SiO_2 substrate (20 %, under white light illumination) and the multi-peak structure of the Raman spectrum (fig. S1a). Following $FeCl_3$

intercalation (*15*), we observe splitting of the G-band into three separate Lorentzian peaks (fig. S1b). Each peak corresponds to a different level of charge carrier concentration due to a specific stage of intercalation (*15*). The G_0 peak at $\sim 1585\ \mathrm{cm}^{-1}$ corresponds to a pristine graphene layer, the G_1 peak at $\sim 1610\ \mathrm{cm}^{-1}$ to a graphene layer in contact with one layer (stage-2) and the G_2 peak at $\sim 1625\ \mathrm{cm}^{-1}$ to a graphene sheet sandwiched between two layers (stage-1). Hence, from the Raman spectrum we can identify the configuration reported in fig. S1c. Here we have one graphene layer which remains isolated from $FeCl_3$. Two graphene layers are in contact with a single layer of intercalant and a fourth graphene layer at the centre of the structure is fully intercalated. It is highly improbable for $FeCl_3$ to remain on the top (or at the bottom) of the flake considering that any such layer would be directly exposed to all solvents used during subsequent device fabrication processes. Furthermore, the G_1 peak intensity is indicative of a larger presence of stage-2 intercalated states, relative to stage-1, as expected for the structure shown in fig. S1c.

S1.2 Exposure time and laser power effect

In order to calibrate the laser-induced displacement of $FeCl_3$ with respect to the incident laser power and time, we performed a Raman spectroscopy study on two spots of a representative flake (shown in fig. S2). The effect of exposing $FeCl_3$-FLG to laser powers of $0.15\ MW/cm^2$, $1.5\ MW/cm^2$, $4.1\ MW/cm^2$ and $15.3\ MW/cm^2$ is shown in fig. S2a to c: it is evident that a change in G_2-peak height, indicative of a reduction in doping, only occurs upon exposure to a high-power light source. The dependence upon time was examined by irradiating a spot on the flake with a fixed power of $15.3\ MW/cm^2$ for 0, 10 and 600 seconds (fig. S2b to d). We observe that the doping modification happens very quickly, within the first 10 seconds, while a prolonged exposure causes no further effect (notably, the defect-related D-peak at $\sim 1350\ \mathrm{cm}^{-1}$ does not emerge). Optical micrographs of the flake before and after laser exposure are shown in fig. S2e, no visible modifications to $FeCl_3$-FLG are observed.

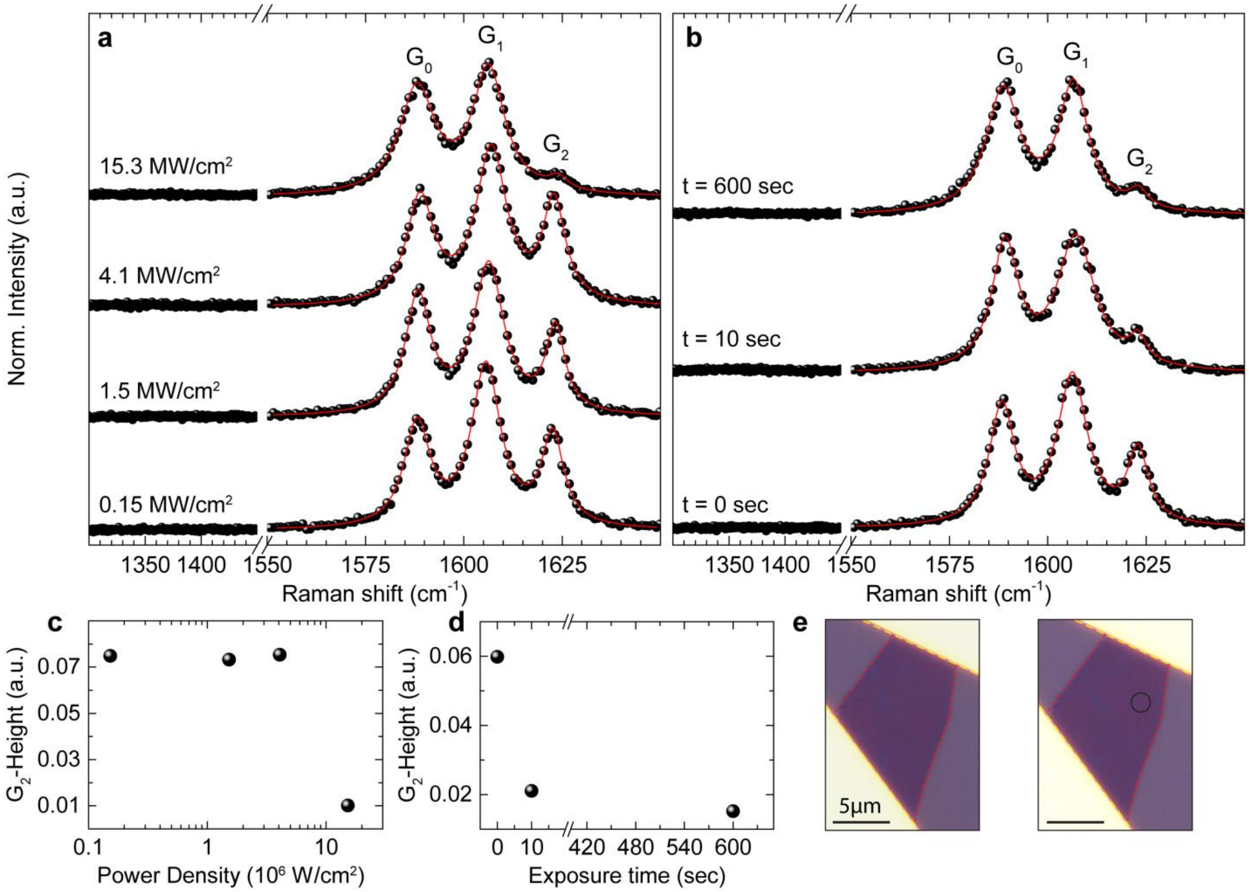

fig. S2. Calibration of laser-induced displacement of $FeCl_3$. (**a**) Raman spectra of $FeCl_3$-FLG acquired on the same location after irradiating with a $532\ nm$ laser light at different incident powers ($0.15\ MW/cm^2$, $1.5\ MW/cm^2$, $4.1\ MW/cm^2$ and $15.3\ MW/cm^2$) for 20 seconds. (**b**) Raman spectra of $FeCl_3$-FLG after irradiating with a power of $15.3\ MW/cm^2$ for 10 and 600 seconds compared with not-irradiated ($t = 0$ seconds). Each spectrum is acquired with the same laser at power of $0.15\ MW/cm^2$, red solid lines are Lorentzian fits. (**c** and **d**) Summary of the G_2-peak Height (normalized to the Si peak at $520\ cm^{-1}$) versus incident power and exposure time, as extrapolated from the fits in panels (a and b). (**e**) Optical micrograph of the examined $FeCl_3$-FLG flake before (right) and after (left) laser irradiation on the highlighted spot (black circle), no optical modifications are visible in the flake.

section S2. Supplementary photocurrent measurements

S2.1 Bandwidth of $FeCl_3$-FLG photodetectors

In fig. S3a we show the frequency-modulated photoresponse of the device presented in Fig. 3a in the main text. The $-3\ \mathrm{dB}$ cut-off gives an operating bandwidth of $700 \pm 5\ \mathrm{Hz}$, in good agreement with the rise and fall time measurements shown in fig. S3b to c.

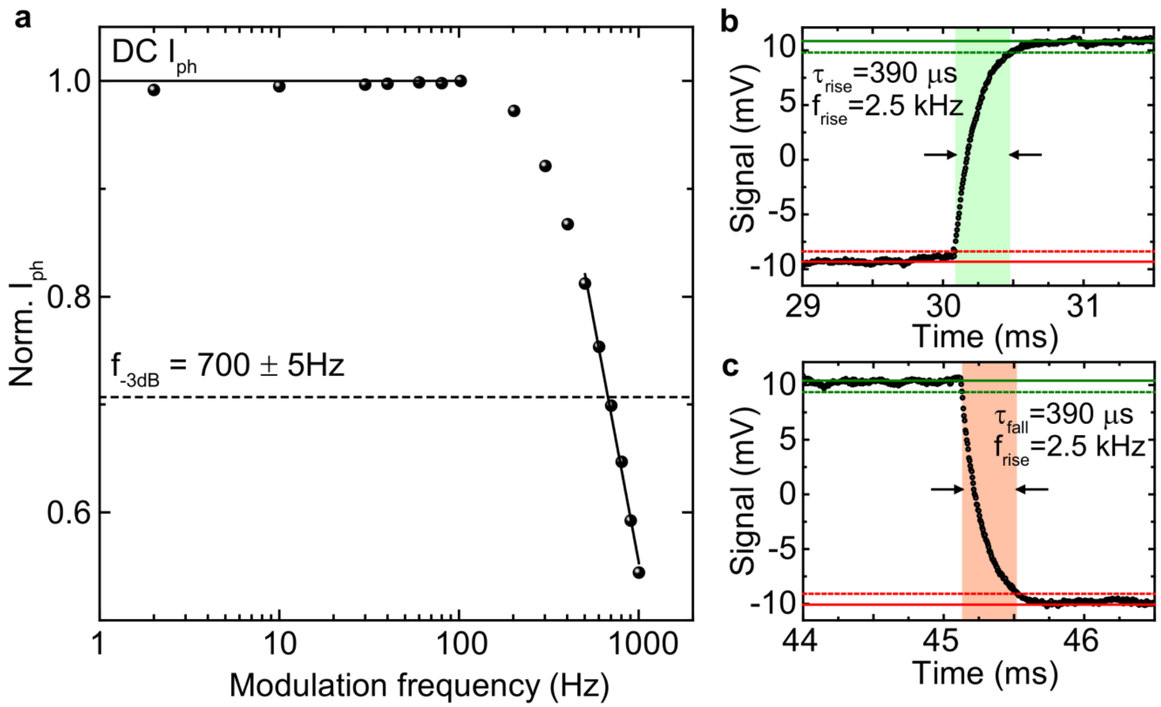

fig. S3. Bandwidth of a laser-written $FeCl_3$-FLG junction device. (**a**) Frequency-modulated photoresponse of the device shown in Fig. 3a, main text: photocurrent is normalized to the DC value and the $-3\ dB$ cut-off is marked by the dashed line. (**b**) Rise and (**c**) fall time of the same device. Solid lines mark the steady state, dashed lines mark the $10\% - 90\%$ thresholds.

S2.2 Noise equivalent power (NEP) measurement

RMS noise measurements were performed with a lock-in amplifier measuring the photocurrent directly with no current preamplifier in the circuit. The lock-in noise equivalent bandwidth (NEBW) was set to be $16.6\ \mathrm{Hz}$, the modulation frequency was

$689\ \mathrm{Hz}$. Measured values are reported in fig. S4 together with values of the photocurrent, as a function of incident laser power. The NEP is extrapolated to be $4\ \mathrm{kW/cm^2}$.

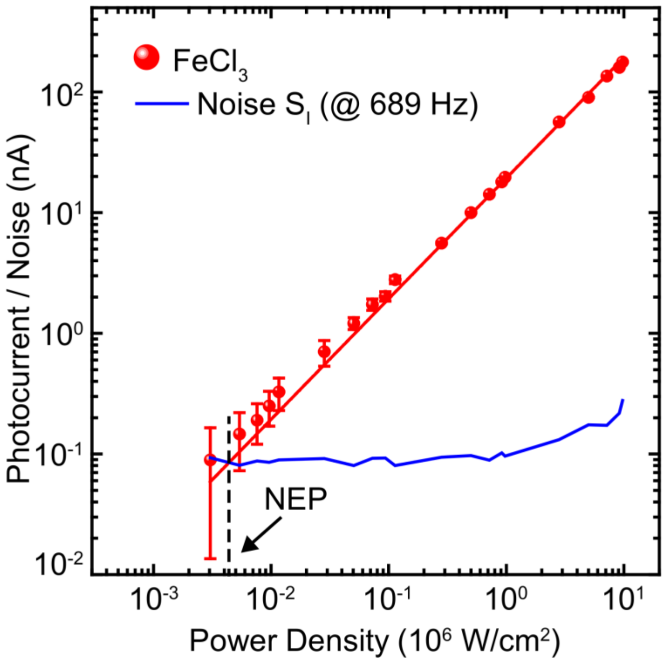

fig. S4. NEP of laser-written FeCl3-FLG junction device. Photoresponse as a function of laser power (red) together with the RMS noise measured during the same experiment (blue). The intersection marks the value of the NEP.

S2.3 Comparison of the LDR of graphene photodetectors

In table S1, we show the saturation power density (P_{sat}) of graphene and functionalized graphene photodetectors reported in literature compared to the values measured in this work for $FeCl_3$-FLG junctions. Previous works have shown deviation from linear behaviour and saturation of photocurrent for power densities $< 57\ \mathrm{kW/cm^2}$ in graphene (*9*) and $< 120\ \mathrm{kW/cm^2}$ in functionalized graphene (*14*). In contrast, $FeCl_3$-FLG junctions show a saturation level $> 10^4\ \mathrm{kW/cm^2}$, more than two orders of magnitude larger than other reports.

In the same table we report the linear dynamic range (LDR) in decibels (dB), calculated as

$$LDR = 10 \times log_{10}\left(\frac{P_{sat}}{NEP}\right)\ [dB] \tag{S1}$$

where the Noise Equivalent Power (NEP) is defined as the power at which the signal to noise ratio (SNR) has a value of 1. The NEP can be measured directly or computed as

$$NEP = \frac{S_I}{R}\left[\frac{W}{\sqrt{Hz}}\right] \tag{S2}$$

table S1. LDR of graphene and functionalized graphene devices.

Literature Reference	P_{sat} [a]	NEP [b]	LDR [c]
Kim *et al.* (*17*)	$10^{-3}\ \mathrm{W/cm^2}$	-	-
Liu *et al.* (*20*)	$1.27\ \mathrm{W/cm^2}$	$0.03\ \mathrm{W/cm^2}$	15 dB [d]
Tielrooij *et al.* (*18*)	$23\ \mathrm{kW/cm^2}$	-	-
Mueller *et al.* (*3*)	$51\ \mathrm{kW/cm^2}$	$10\ \mathrm{kW/cm^2}$	7.5 dB [e]
Graham *et al.* (*9*)	$57\ \mathrm{kW/cm^2}$	-	-
Patil *et al.* (*19*)	$14\ \mathrm{kW/cm^2}$	-	-
Wang *et al.* (*13*)	$120\ \mathrm{kW/cm^2}$	$3.3\ \mathrm{kW/cm^2}$	15 dB [e]
This work (Graphene)	$45\ \mathrm{kW/cm^2}$	-	-
**This work ($FeCl_3$-FLG)**	$\mathbf{> 10^4\ kW/cm^2}$	$\mathbf{4\ kW/cm^2}$	**44 dB** [d]

[a] Power density at which saturation of photocurrent is observed; [b] Noise Equivalent Power; [c] Linear Dynamic Range; [d] Measured; [e] Estimated.

where S_I is the RMS current noise (in $A/\sqrt{\mathrm{Hz}}$) and R is the responsivity of the photodetector (in $\mathrm{A/W}$). We used equation S2 to calculate the NEP of different graphene-based photodetectors reported in literature (*9,13*). Assuming a graphene

photodetector operating at the same frequency as our device ($689\ \mathrm{Hz}$, see section S2.1), we can assume that the main source of noise will be the $1/f$ contribution (*35*). Using the results in references (*35*) and (*36*) we assume a spectral noise of $S_I = 1.0 \times 10^{-8}\ A/\sqrt{\mathrm{Hz}}$. The NEP for reference (*20*) is taken from the measured values, the LDR agrees well with our estimation for the other references.

S2.4 Photocurrent in pristine graphene

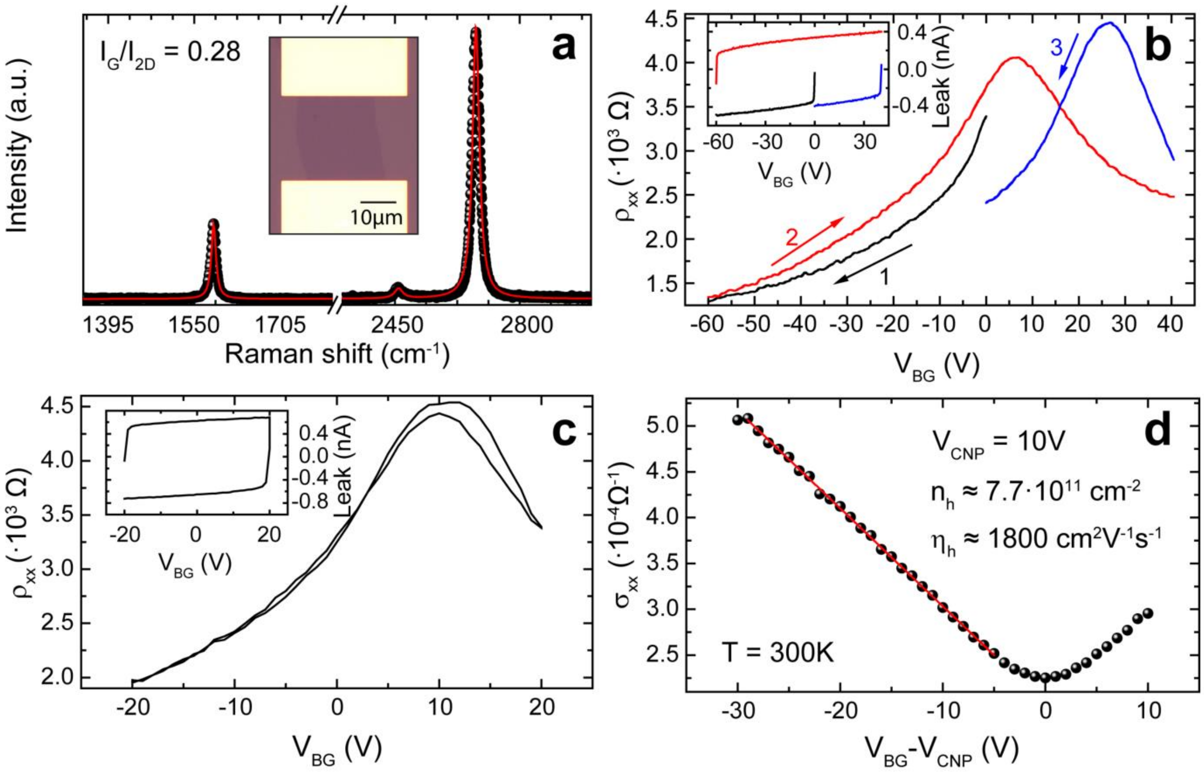

fig. S5. Characterization of supported pristine graphene devices. (**a**) Raman spectrum of a monolayer graphene device. Inset: Optical micrograph of the same sample. (**b**) Longitudinal resistivity (ρ_{xx}) as a function of gate voltage (V_{BG}) for the device shown in panel (**a**) before ~20 hours in Acetone ($60\ °C$) and rinsing for 1 hour in Isopropanol ($60\ °C$). Numbers indicate the chronological sequence of gate voltage sweeps. (**c**) Gate sweeps of the same device after acetone-IPA treatment. Insets: gate leakage current as a function of gate voltage. (**d**) Conductivity (σ_{xx}) as a function of $V_{BG} - V_{CNP}$ with the extrapolated values for the charge concentration and mobility. All measurements are performed at room temperature in air.

Measurements shown in Fig. 3a and b of the main text (black dots) were performed on a pristine graphene device consisting of a monolayer flake mechanically-exfoliated onto p-doped Si with a 280 nm surface oxide. Cr/Au ($5/50$ nm respectively) electrodes were defined via electron-beam lithography using a PMMA resist followed by thermal evaporation of the metals and lift-off in Acetone. Figure S5a shows a representative Raman spectrum and optical micrograph of the resultant device. We fit both the G and 2D bands with a single Lorentzian, revealing a relative intensity of $I_G/I_{2D} = 0.28$. The optical contrast between the graphene and Si/SiO_2 substrate is 5% which, combined with a non-degenerate 2D band and $I_G/I_{2D} < 1$, signifies the presence of a graphene monolayer. Figure S5b shows the longitudinal resistivity (ρ_{xx}) as a function of back-gate voltage (V_{BG}) for the same device. From an initial gate sweep, the charge-neutrality point (V_{CNP}) is located around 0 V. However, a large hysteresis is observed during subsequent sweeps with a shift in V_{CNP} by as much as 30 V. This behaviour is typical of graphene devices with a high degree of surface contamination (e.g. polymer residues from fabrication) measured in atmosphere. Surface contaminants induce charge-transfer which affects the capacitive gating effect (*37*). To minimise the effect of impurities on the surface of graphene, we soaked this device in warm Acetone (60 °C) for ~20 hours and then rinsed in warm Isopropanol for 1 hour. The gate response following this procedure is shown in fig. S5c where hysteresis effects are greatly reduced, resulting in a stable neutrality point at $V_{CNP} = 10V$. We extract the hole concentration (n_h) and field-effect mobility (η_h) of our device using the relationships $n_i = \epsilon V_{CNP}/et$ and $\eta_i = \sigma/en_i$, where i indicates the polarity of charge carriers, e is the electron charge, t and ϵ are the thickness and absolute permittivity of respectively. The resulting values, shown in fig. S5d, are $n_h \approx 7.7 \cdot 10^{11}\ \mathrm{cm}^{-2}$ and $\eta_h \approx 1800\ \mathrm{cm}^2\mathrm{V}^{-1}\mathrm{s}^{-1}$. Having reduced the charge carrier concentration two orders of magnitude below that of $FeCl_3$-FLG layers, we performed the photo-current measurements shown in Fig. 3a (main text) at $V_{BG} = 0V$.

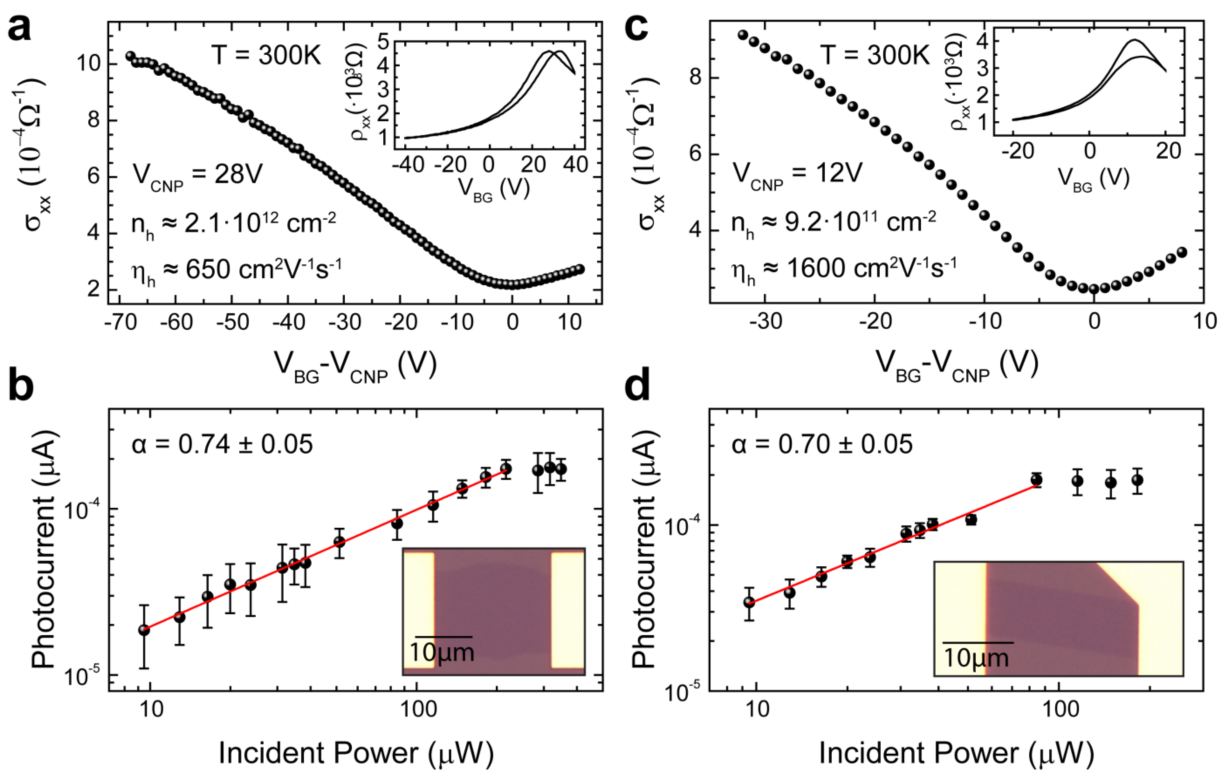

fig. S6. Additional measurements of photocurrent in supported pristine graphene devices. (**a**) Conductivity (σ_{xx}) as function of gate voltage relative to the charge neutrality point device A. Inset, gate voltage dependence of resistivity. (**b**) Photo-current as function of laser incident power ($\lambda = 473\ nm$, $V_{BG} = 0\ V$) for device A. Inset, micrograph of device A. Equivalent measurements for device B are shown in panels (**c**) and (**d**). Measurements were taken in ambient conditions and at room temperature after prolonged soaking in warm acetone and Isopropanol (see fig. S5).

Figure S6 shows the electrical and optoelectronic characterisation of two other pristine graphene devices (A and B respectively). Measurements were performed in ambient conditions after soaking each device in acetone for ~20 hours. Figure S6a and c show marginal differences in carrier concentration due to surface contamination. The power-dependence of the photocurrent ($I_{ph} \propto P_{opt}^{\alpha}$) measured in samples A (fig. S6b) and B (fig. S6d) were taken with a $\lambda = 473$ nm excitation laser, $V_{BG} = 0$ V and 10 mV applied between source and drain. Power-law exponents of $\alpha = 0.74 \pm 0.05$ and $\alpha = 0.70 \pm 0.05$

were extracted, both in agreement with dominant photothermoelectric effects observed in supported pristine graphene devices.

S2.5 Photocurrent at p-p’ junctions in $FeCl_3$-FLG

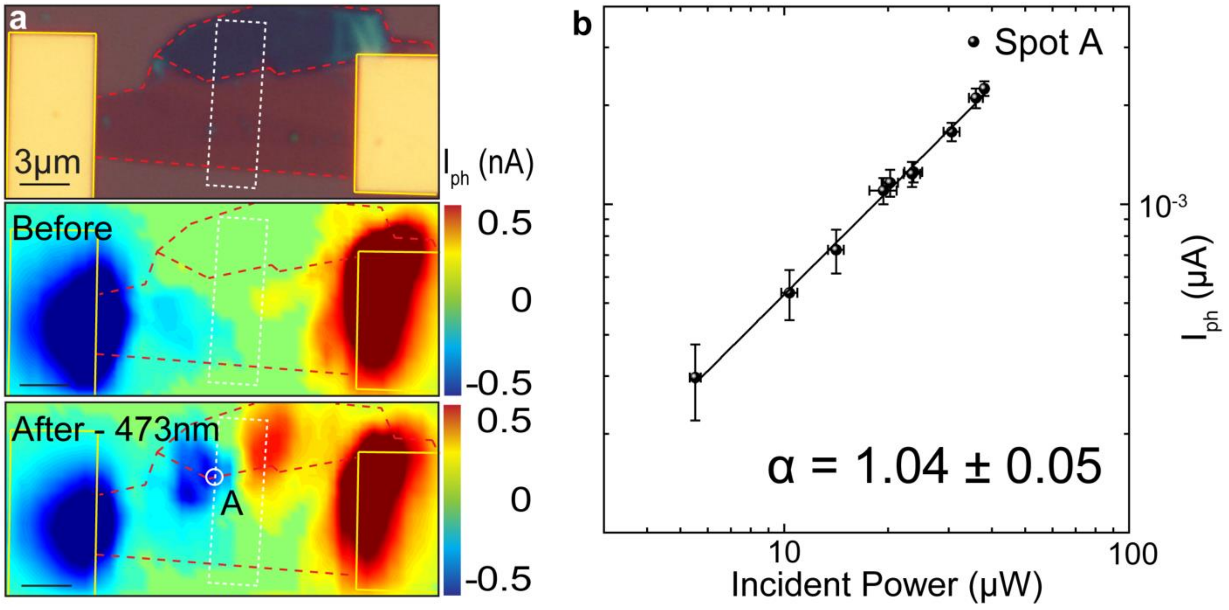

fig. S7. Photoresponse at p-p′ junction in FLG. (**a**) Optical micrograph (top panel) and scanning photocurrent maps of a $FeCl_3$-FLG flake before (middle panel) and after (bottom panel) laser-induced de-intercalation. Superimposed lines indicate boundaries of the $FeCl_3$-FLG flake (red dashes), Au contacts (yellow) and de-intercalated area (white-dashes). Scale bars, $3\ \mu m$. (**b**) Absolute photocurrent as a function of incident power measured at spot A (white circle in **a**) for $\lambda = 473\ nm$ excitation, a power exponent of $\alpha = 1.04 \pm 0.05$ is obtained from a fit to the experimental data (solid line).

Figure S7 presents photocurrent measurements at p-p’ interfaces of $FeCl_3$-FLG in addition to those shown in the main text. All measurements were taken in short-circuit configuration with a two terminal device geometry. An optical micrograph image of the $FeCl_3$-FLG flake is shown in fig. S7a where two distinct areas of different thickness are apparent. No substantial photocurrent is observed between these two regions either before or after laser-induced de-intercalation. After performing a raster scan with a $15.3\ \mathrm{MW/cm^2}$ incident laser power ($\lambda = 532\ \mathrm{nm}$, $1\ \mu\mathrm{m}$ steps) over the region highlighted

by the white dashed line, photocurrent was measured at the p-p' interfaces. The power dependence of this photocurrent (fig. S7b) exhibits an exponent of $\alpha = 1.04 \pm 0.05$, similar to measurements shown in Fig. 3a and b of the main text.

section S3. Power dependence of the photothermoelectric and photovoltaic effects

S3.1 Power dependence of the photothermoelectric (PTE) effect

The photothermoelectric (PTE) effect can exhibit a variety of power law ($I_{ph} \propto {P_{opt}}^{\alpha}$) exponents depending on the dominant cooling mechanism and the average temperature of hot carriers (T_h) relative to that of the lattice/environment (T_l). This is due to presence of a "bottleneck effect" whereby T_h may remain above T_l for photo-excited carriers in graphene due to the limited availability of pathways for heat dissipation. Initial coupling with high-energy optical phonon modes is exhausted for chemical potential (μ) $< 200\ \mathrm{meV}$, leaving hot carriers to equilibrate through electron-electron scattering then gradually lose energy to the lattice (*38*). Heat dissipation is slow due to the small Fermi surface of graphene which limits energy losses through the momentum-conserving emission of an acoustic phonon ($\Delta E_{ac} < 2\hbar v_s\, k$ where $v_s \sim 2 \cdot 10^{\,4}\ \mathrm{ms}^{-1}$ is the acoustic phonon speed (*39*) and k is the hot-carrier wavenumber) (*23*). The "supercollision" model (*9,24*) recognises that, in this situation, short-range scattering at sites of disorder allow a far larger transfer of energy and will be the dominant mechanism of carrier relaxation. The rate of heat loss (H) when supercollisions are dominant is given by

$$H_{SC} = A\left({T_h}^3 - {T_l}^3\right), \qquad A = \frac{9.62 g^2 D(\mu)^2 k_B^3}{\hbar k l} \tag{S3}$$

where g is the electron-phonon coupling frequency, $D(\mu)$ is the density of states and l is the mean free path of hot carriers. Under continuous wave (CW) illumination, a steady-state is reached when the optical power imparted to hot carriers equals the power transferred to the lattice ($P_{in} = H_{SC}$). The electron temperature may be related to the photothermoelectric current using the Mott relation (*27*)

$$S = -\frac{\pi^2 k_B^2 T_h}{3e} \cdot \frac{1}{\sigma} \cdot \frac{\partial \sigma}{\partial \mu} \tag{S4}$$

in conjunction with a general expression for the photothermoelectric voltage generated at the junction of two materials, $V_{PTE} = (S' - S)\Delta T$, to give (*5*)

$$I_{PTE} = \beta T_h (T_h - T_l) \tag{S5}$$

where

$$\beta = -\frac{\pi^2 {k_b}^2}{3e}\left[\frac{1}{\sigma'} \cdot \frac{d\sigma'}{d\mu'} - \frac{1}{\sigma} \cdot \frac{\partial \sigma}{\partial \mu}\right] \tag{S6}$$

Assuming that hot electrons stabilise at a temperature far above that of the lattice ($T_h > > T_l$), equation (S3) may be reduced to

$$T_h = (P_{in}/A)^{1/3} \tag{S7}$$

Similarly, equation (S5) becomes

$$I_{PTE} = \beta T_h^2 \tag{S8}$$

Hence, the measured photocurrent should have a power dependence of

$$I_{PTE} = \beta \left(\frac{P_{in}}{A}\right)^{2/3} \tag{S9}$$

This is the power exponent commonly measured in graphene photodetectors on Si/SiO_2 substrates.

In the case where the electron temperature is only marginally above that of the environment ($T_h - T_l << T_l$, as is common for measurements in CW illumination (*40*)) a Taylor expansion of equation (S3) about $T_h \approx T_l$ yields

$$P_{in} \approx 3AT_l^{\ 2}(T_h - T_l) \tag{S10}$$

Combining equation (S10) with equation (S5), we find an approximately linear dependence between photocurrent and power

$$I_{PTE} = \frac{\beta P_{in}}{3AT_l} + \frac{\beta P_{in}^{\ 2}}{9A^2T_l^{\ 4}} \approx \frac{\beta P_{in}}{3AT_l} \tag{S11}$$

Table S2 compiles the power-law exponents obtained from equivalent calculations using models which base $P_{in}(T_h)$ purely upon acoustic phonon scattering (*9,24*). All models of the PTE effect predict an approximately linear dynamic range when $T_h \approx T_l$, this condition is most likely to be satisfied by measuring I_{PTE} at room temperature and with low incident powers.

The relative contributions of acoustic phonon scattering (H_{AP}) and supercollisions (H_{SC}) to the rate of heat loss from photo-excited charge carriers is determined by the degree of disorder in the sample, the environmental temperature and the size of the Fermi surface (i.e. the level of doping) (*24*)

$$\frac{H_{SC}}{H_{AP}} = \frac{0.77}{kl}\frac{\left(T_h^{\ 2} + T_hT_l + T_l^{\ 2}\right)}{T_{BG}^{\ 2}} \tag{S12}$$

Equation (S12) is valid when $k_BT \ll \epsilon_F$, where ϵ_F is the Fermi level. T_{BG} is the Bloch-Grüneisen temperature of graphene (*26,41*) ($T_{BG} = \Delta E_{ac}/k_B$). The degree of disorder and doping will vary significantly between samples and therefore makes the wide variation in power dependence characteristics reported for graphene photodetectors understandable. In the case of $FeCl_3$-FLG, high levels of p-doping will significantly increase the Fermi surface thereby allowing larger energy losses via momentum-conserving acoustic phonon emission. As a result, hot carrier bottleneck effects will be less prominent and the contribution of defect-assisted scattering towards photocurrent in $FeCl_3$-FLG is likely to be small compared to interfaces in graphene photodetectors with low levels of doping.

table S2. Summary of power-law exponents possible for photocurrent originating from the photothermoelectric effect.

PTE Model	$\mathbf{P_{in}}(\mathbf{T_e})$	$\alpha\ (T_e \gg T_l)$	$\alpha\ (T_e - T_l \ll T_l)$
**Supercollision**	$A\left({T_e}^3 - {T_l}^3\right)$	2/3	≈ 1
**Acoustic** $(k_B T >> \epsilon_F)$	$A' {T_e}^4 (T_e - T_l)$	2/5	≈ 1
**Acoustic** $(k_B T << \epsilon_F)$	$A''(T_e - T_l)$	2	≈ 1

S3.2 Power dependence of the photovoltaic (PV) effect

The photovoltaic effect describes the separation of an electron-hole pair by an in-built electric field. In the low-power regime where the photocarrier lifetime, τ_c, is independent of the photo-generation rate, r_g, photocurrent may be shown to have a linear dependence upon incident power, with $I_{PVE} \propto P_{opt}$. For a photodetection layer the steady-state photo-generation rate of carriers is given by (*25*)

$$r_g = \frac{\chi \Phi_{ph}}{A_{ph} D} \tag{S13}$$

where χ is the quantum efficiency of the absorption process, Φ_{ph} is the incident photon flux, A_{ph} is the illuminated area and D is the thickness of the layer. The recombination rate of excess carriers depends on the minority carrier lifetime τ_c via

$$r_r = \frac{n}{\tau_c} = \frac{p}{\tau_c} \tag{S14}$$

where n and p are the excess carriers populations. Therefore, in equilibrium the generation rate must equal the recombination rate and the photocarrier density is

$$n = p = r_g \tau_c = \frac{\chi \Phi_{ph} \tau_c}{A_{ph} D} \quad \text{(S15)}$$

Given a potential difference V, between the sides of the layers, a photoinduced current I_{PVE} can be measured

$$I_{PVE} = \frac{WD}{L} \sigma V = \frac{WD}{L} r_g \tau_c e(\eta_e + \eta_h) V \quad \text{(S16)}$$

where $\sigma = ne\eta_e + pe\eta_h$ is the electrical conductivity, η_h and η_e are the hole and electron mobilities, W and L are the width and the length of the channel and e is the electron charge. Combining equation (S15) and equation (S16) and noting that $\Phi_{ph} = P_{opt}/h\nu$, where P_{opt} is the incident optical power, h is Plank's constant and ν is the frequency of the incident light, we arrive to the final expression

$$I_{PVE} = \eta \frac{P_{opt}}{h\nu} \frac{e(\eta_e + \eta_h) V \tau_c W}{A_{ph} L} \quad \text{(S17)}$$

Hence we can define the photoconductive gain G as the ratio of the rate of flow of electrons per second from the device to the rate of generation of e-h pairs within the device

$$G = \frac{I_{PVE}}{e} \frac{1}{r_g WDL} = \frac{\tau_c (\eta_e + \eta_h) V}{L^2} \quad \text{(S18)}$$

Equation (S17) shows the relation $I_{PVE} \propto (P_{opt})^{\alpha}$ with $\alpha = 1$.

section S4. Estimation of chemical potential and conductivity for decoupled graphene layers

S4.1 Estimation of chemical potential

In order to explain the physical mechanisms responsible of the measured photoresponse at p-p' junctions in $FeCl_3$-FLG, it is necessary to estimate the chemical potential of an intercalated flake before and after laser irradiation. Previous studies have shown through Raman spectroscopy (*42*) and magneto-transport measurements (*14*) that highly intercalated samples of $FeCl_3$-FLG may be considered as parallel stacks of

electrically isolated monolayers. Using the density of states for monolayer graphene, we define the chemical potential (μ) of each decoupled graphene sheet as $\mu = \hbar v_F \sqrt{\pi n}$ where $v_F \approx 10^6 \text{ ms}^{-1}$ is the Fermi velocity and n is the density of holes. Note that we equate the chemical potential of our system with the Fermi level, as $k_B T \ll \epsilon_F$ for all situations relevant to our discussion. Following the reasoning given in section S1.1, the flake shown in fig. S8a is four-layer graphene the bottom two sheets remain electrically coupled as a bilayer (fig. S8b). Going from top to bottom, we now refer to the decoupled graphene systems as A, B and C (fig. S8b). Dash terms (e.g. σ_A') represent a material's properties after laser-induced de-intercalation.

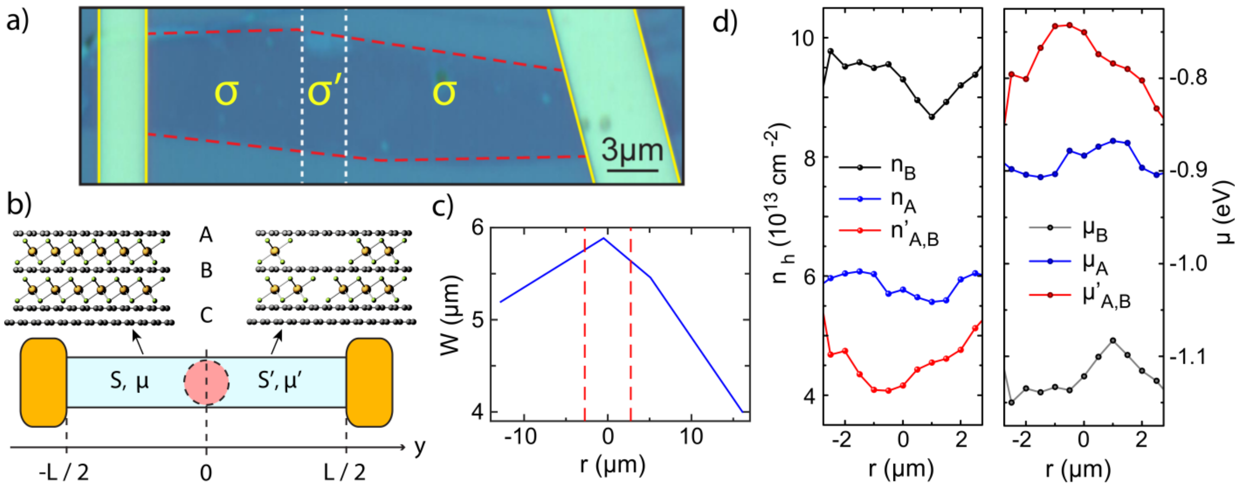

fig. S8. Calculation of the carrier concentration and chemical potential at p-p′ interfaces of $FeCl_3$-FLG. (**a**) Micrograph picture of a four-layer $FeCl_3$-FLG flake with a p-p'-p junction patterned by $\lambda = 532\, nm$ laser irradiation (main text). Superimposed lines represent boundaries of the flake (red), contacts (yellow) and the de-intercalated area (white). (**b**) Schematic of a p-p' interface located at the centre of a long, narrow $FeCl_3$-FLG channel. The degree of intercalation, inferred from Raman spectroscopy measurements, is illustrated for each region with the three decoupled systems labelled A, B and C. (**c**) Width of the flake shown in (a) as a function of distance along the channel length. Red dashes mark the boundaries of the p' region. (**d**) Concentration of charge carriers in decoupled graphene layers inferred from the position of the G_1 and G_2 Raman peak positions shown in Fig. 1c (main text). The chemical potential is then calculated using the density of states for monolayer graphene.

The chemical potential of the bilayer system C will not be affected as radically as the monolayers A or B when in proximity to 1 layer of $FeCl_3$, we therefore focus our discussion on the upper two layers of the flake. In fig. S8d, the model of Lazzeri *et al.* (*21*) is used to convert from the positions of G_1 and G_2 Raman peaks to the carrier concentration in each layer before and after laser writing ($n'_A \approx n'_B$ after irradiation). Taking a linear band approximation, the respective chemical potentials are plotted in fig. S8d, giving average values of $\mu_A = (-0.88 \pm 0.02)eV$, $\mu_B = (-1.12 \pm 0.2)eV$ and $\mu'_{A,B} = (-0.76 \pm 0.02)eV$. Marginally smaller shifts in Fermi level have been measured in intercalated graphene grown by chemical vapour deposition (*15*), but our estimated values agree well with those previously reported in DFT calculations (*28*) and Raman spectroscopy measurements (*14,42*) of exfoliated flakes.

S4.2 Estimation of conductivity

Two terminal resistance measurements of the $FeCl_3$-FLG flake in fig. S8a were taken before and after laser patterning using a lock-in amplifier in constant current configuration. Through image analysis, we calculate the change in channel width along the entire flake (fig. S8c) and relate it to the conductivity, σ_{tot}, of the two different regions

$$R_{SD} = \frac{1}{\sigma_{tot}} \int_{-L/2}^{L/2} \frac{1}{W(y)} dy \tag{S19}$$

$$R'_{SD} = \frac{1}{\sigma_{tot}} \left[\int_{-L/2}^{y_1} W\,(y)^{-1} dy + \int_{y_2}^{L/2} W\,(y)^{-1} dy \right] + \frac{1}{\sigma'_{tot}} \int_{y_1}^{y_2} W\,(y)^{-1} dy \tag{S20}$$

where y_1 and y_2 denote the boundaries of the irradiated p' area. Through equations (S19) and (S20) we find $\sigma_{tot} \approx 27\ \mathrm{m}S$ and $\sigma'_{tot} \approx 10\ \mathrm{m}S$, slightly below the maximum conductivity of fully intercalated four-layer flakes.[14] Approximating $n_{tot} \approx 2n_A + n_B$ and $n'_{tot} \approx 3n'_{A,B}$, the average hole mobility is taken to be $\langle\eta\rangle = 650\ \mathrm{cm^2V^{-1}s^{-1}}$. Lastly, we attain conductivity values for the individual systems A and B using

$$\sigma(\mu) = \frac{e\eta\mu^2}{\pi\hbar^2 v_F^2} + \sigma_{min} \tag{S21}$$

where $\sigma_{min} \sim 4e^2/h$.[43] This may also be written in the form

$$\sigma(\mu) = \sigma_{min}\left(1 + \frac{\mu^2}{\Lambda^2}\right), \quad \Lambda \approx 140meV \tag{S22}$$

We find $\sigma_A = 6.0\ \mathrm{mS}$, $\sigma_B = 9.6\ \mathrm{mS}$ and $\sigma'_{A,B} = 4.5\ \mathrm{mS}$.

section S5. Physical explanation for a purely photovoltaic response

Here, we estimate the relative magnitudes of photocurrent produced by the photovoltaic and photothermoelectric effects at a p-p' junction of $FeCl_3$-FLG. We consider a single junction located in the middle of an $FeCl_3$-FLG channel (fig. S8b) in order to simplify our explanation of the underlying photoresponse mechanisms and demonstrate that the suppression of thermoelectric currents in our devices is not simply due to the proximity of two junctions with opposing polarity. Following a similar method to Song *et al.* (*5*), the total photocurrent produced when the interface is illuminated, under short circuit conditions, is taken to be a summation of photovoltaic and thermoelectric contributions

$$I_{PH} = \frac{1}{RW}\int_0^W \int_{-L/2}^{L/2} \left[S(x,y)\nabla T(x,y) - \sigma(x,y)^{-1} e\eta n_{ph}(x,y)\nabla U(x,y)\right] dydx \tag{S23}$$

The first term of the integral represents thermoelectric currents produced by a temperature gradient $\nabla T(x,y)$ in a material with a spatially varying Seebeck coefficient $S(x,y)$. The second term describes the photovoltaic response produced when a density $n_{ph}(x,y)$ of carriers are generated in a material and then displaced by an in-built potential gradient $\nabla U(x,y)$.

S5.1 Photothermoelectric Effect (PTE)

Approximating $S(y)$ as a step change at the p-p' junction and substituting equation (S22) into equation (S4), we re-write the PTE current in terms of the electrical properties of the regions either side of the p-p' interface (fig. S8a)

$$I_{PTE} = \frac{2\pi^2 k_B^2 T_h}{3eR} \cdot \frac{\Delta T}{\mu\mu'} \cdot \left[\mu'^{\left(1-\frac{\sigma_{min}}{\sigma}\right)} - \mu\left(1 - \frac{\sigma_{min}}{\sigma'}\right)\right] \qquad (S24)$$

The difference in steady state temperature between the lattice and hot carriers ($\Delta T = T_h - T_l$) is a difficult quantity to measure, requiring picosecond resolution of photocurrent transients in low temperature environments (*9)* which are beyond the scope of our experimental apparatus. Alternative methods which approximate values of ΔT using equation (S24) rely on the assumption that any measured photovoltage is produced solely by thermoelectric currents (*7*). This inference cannot be made for $FeCl_3$-FLG interfaces; extremely high carrier densities (up to $3 \times 10^{14}\ \mathrm{cm}^{-2}$ per layer) efficiently screen electrostatic gating potentials and prohibit experimental methods which are typically used to verify the “six fold pattern” signature of the PTE effect (*5,7,9,40*). Instead, we use a solution obtained for the one-dimensional heat equation of our system, where the photocurrent density created at the p-p' junction is assumed to be a delta function with respect to the laser spot size (*5*)

$$\Delta T = \frac{\alpha \epsilon_0 l_0 N_{ph}}{\frac{\kappa}{\zeta} coth\left(\frac{L}{2\zeta}\right) + \frac{\kappa'}{\zeta'} coth\left(\frac{L}{2\zeta'}\right) + \frac{T_0}{RW}(S' - S)^2} \qquad (S25)$$

Here, α is the fraction of an absorbed photon's energy (ϵ_0) which is retained by the hot electron system once electron-electron interactions and coupling with optical phonons have been exhausted. l_0 is the laser spot diameter and N_{ph} represents the flux of absorbed photons at the centre of the p-p' junction averaged over the channel width. κ and ζ are the thermal conductivity and average cooling length of hot electrons respectively. Provided $k_B T \leq (\mu, \mu', \Delta)$, the third term of the denominator in equation

(S25) is negligible. The cooling length of each graphene layer is dependent upon its electrical conductivity, density of states ($D(\mu)$) and the hot carrier cooling rate (γ) (*7*)

$$\zeta = \sqrt{\frac{\sigma}{\gamma e^2 D(\mu)}} \tag{S26}$$

Naturally, γ is dependent upon the prevailing hot electron scattering mechanism. For graphene layers where $n \geq 10^{13}\ \mathrm{cm}^{-2}$, the Bloch-Grüneisen temperature reaches hundreds of Kelvin and hot electrons may completely equilibrate with the lattice via just a single acoustic phonon interaction under CW illumination (*26*). Disorder-mediated scattering is therefore not relevant in our devices. This can be shown by substituting equation (S21) into equation (S12) using the relation for the mean free path of a non-degenerate two-dimensional electron gas, $l = \sigma\hbar\pi/ke^2$, to estimate the relative magnitudes of power loss via supercollisions and momentum-conserving scattering events in $FeCl_3$-FLG. For $T_h - T_l \ll T_l$, we find supercollisions to make up as little as 3% (11%) of the total heat loss from hot electrons before (after) laser-induced de-intercalation. The scattering rate can therefore be approximated by considering just single acoustic phonon processes (*23*) as

$$\gamma = \frac{3D^2\mu^3}{4\pi^2\hbar^3\rho_m v_F^4 k_B T_{el}} \tag{S27}$$

where $D \sim 20\ \mathrm{eV}$ is the typical screened deformation potential on Si/SiO_2 substrates (*26*) and $\rho_m = 7.6 \cdot 10^{-7}\ \mathrm{kg\ m^{-2}}$ is the mass density of monolayer graphene. Due to the doping induced by $FeCl_3$ intercalation, the cooling rate of momentum-conserving acoustic phonon coupling dramatically increases from $\gamma \sim 10^{-9}\ \mathrm{s^{-1}}$ at $\mu = 100\ \mathrm{meV}$ to $\gamma_A = 6 \cdot 10^{11}\ \mathrm{s^{-1}}$, $\gamma_B = 1 \cdot 10^{12}\ \mathrm{s^{-1}}$ and $\gamma'_{A,B} = 4 \cdot 10^{11}\ \mathrm{s^{-1}}$. This is in agreement with the picosecond relaxation time-scales of $FeCl_3$-FLG measured via pump-probe spectroscopy (*28*). Hence, we use equation (S26) to calculate cooling lengths of $\zeta_A = 220\ \mathrm{nm}$, $\zeta_B = 170\ \mathrm{nm}$ and $\zeta'_{A,B} = 260\ \mathrm{nm}$. Given that $\zeta \ll L/2$ for all of our devices, equation (S25) simplifies to

$$\Delta T \approx \alpha \epsilon_0 l_0 N_{ph} \left(\frac{\kappa}{\zeta} + \frac{\kappa'}{\zeta'}\right)^{-1} \quad (S28)$$

Substituting equation (S28) into equation (S24) and employing the Wiedemann-Franz relation (*44*), we arrive at a full expression for the photothermoelectric current produced at a p-p' junction in $FeCl_3$-FLG

$$I_{PTE} = \frac{2eqk_B T_{el} l_0 N_{ph}}{\mu \mu' R} \cdot \left[\mu'\left(1 - \frac{\sigma_{min}}{\sigma}\right) - \mu_1\left(1 - \frac{\sigma_{min}}{\sigma'}\right)\right] \cdot \left[\frac{\sigma}{\zeta} + \frac{\sigma'}{\zeta'}\right]^{-1} \quad (S29)$$

where $q \sim \alpha \epsilon_0 / k_B T_{el}$ is the internal quantum efficiency.

S5.2 Photovoltaic Effect (PVE)

From equation (S23), the photovoltaic contribution to the photocurrent is

$$I_{PVE} = -\frac{1}{RW} \int_0^W \int_{-\frac{L}{2}}^{\frac{L}{2}} \sigma\,(x,y)^{-1} e \eta n_{ph}(x,y) \nabla U(x,y) dy dx \quad (S30)$$

Taking all values as averages over the channel width, $e\nabla U(y) = \nabla \mu(y)$ and using equation (S22), equation (S30) may be simplified as

$$I_{PVE} = -\frac{\eta\, n_{ph\,(y=0)}}{\sigma_{min} R} \int_{-\frac{L}{2}}^{\frac{L}{2}} \nabla\, \mu(y) \cdot \left(1 + \frac{\mu(y)^2}{\Lambda^2}\right)^{-1} dy \quad (S31)$$

By changing variables, we find a complete expression for the photovoltaic contribution to photocurrent

$$I_{PVE} = \frac{q N_{ph} \eta \Lambda}{\sigma_{min} R \langle\gamma\rangle} \cdot \left[tan^{-1}\left(\frac{\mu}{\Lambda}\right) - tan^{-1}\left(\frac{\mu'}{\Lambda}\right)\right] \quad (S32)$$

Here, we have approximated the steady state density of photogenerated carriers at the p-p' junction as $n_{ph\,(y=0)} \approx q N_{ph} / 2\langle\gamma\rangle$ where $\langle\gamma\rangle$ is the average cooling rate of hot

carriers over both sides of the p-p' junction and the average lifetime of a photogenerated carrier is $\tau \sim \langle\gamma\rangle^{-1}$.

S5.3 Relative magnitudes of the PTE and PVE

Dividing equation (S29) by equation (S32), the relative magnitudes of photothermoelectric and photovoltaic currents at $FeCl_3$-FLG p-p' junctions may be calculated

$$\frac{I_{PTE}}{I_{PVE}} = \frac{2ek_B T_{el} l_0 \langle\gamma\rangle}{\eta\Lambda} \cdot \frac{\left[\mu'\left(1-\frac{\sigma_{min}}{\sigma}\right) - \mu_1\left(1-\frac{\sigma_{min}}{\sigma'}\right)\right]}{\mu\mu'\left(\frac{\sigma}{\zeta}+\frac{\sigma'}{\zeta'}\right)\left[tan^{-1}\left(\frac{\mu}{\Lambda}\right) - tan^{-1}\left(\frac{\mu'}{\Lambda}\right)\right]} \qquad \text{(S33)}$$

For both decoupled systems A and B we calculate $I_{PTE}/I_{PVE} \approx -0.06$, hot carrier dynamics therefore make a negligible contribution to the total photocurrent generated at $FeCl_3$-FLG p-p' junctions and act in the opposite direction to currents produced by the photovoltaic effect.

S5.4 Direction of photocurrent at p-p' junctions in $FeCl_3$-FLG

Based upon previously reported theoretical models (*5*) for graphene-based photodetectors with split electrostatic gates and equation (S33), photothermoelectric currents in graphene will travel in the opposite direction to photovoltaic currents at p-p' and n-n' junctions. This is due to the additional polarity change which PTE currents undergo which is often illustrated by the "six fold pattern" of photocurrent measured at dual-gated interfaces (*7,9,40*). Taking advantage of this asymmetry, we examine the direction of the photocurrent measured at p-p'-p junctions in order to further confirm that the PVE is indeed dominant in laser-written $FeCl_3$-FLG photodetectors.

Figure S9a shows a scanning photocurrent map taken from the main text of a laser-irradiated $FeCl_3$-FLG flake with a p-p'-p junction. This measurement was performed with source and drain electrodes grounded and a current amplifier (DL Instruments, Model 1211) connected in series with the left electrode which sends an output voltage signal, V_{OUT}, to a lock-in amplifier. Calibrating this measurement circuit with a known DC voltage input, we find that positive (red) photocurrent in fig. S9a signifies the drift of

holes to the right electrode and electrons to the left. If hot carrier dynamics are suppressed, photocurrent at laser-written interfaces of $FeCl_3$-FLG will flow in the direction illustrated in fig. S9b, where charges drift with respect to the local potential gradient. However, if PTE effects dominate the measured photoresponse then the configuration illustrated in fig. S9c is expected. Comparing fig. S9a with each of these two scenarios, it is clear that the photocurrent measured at p-p'-p interfaces of $FeCl_3$-FLG is predominantly produced by the photovoltaic effect.

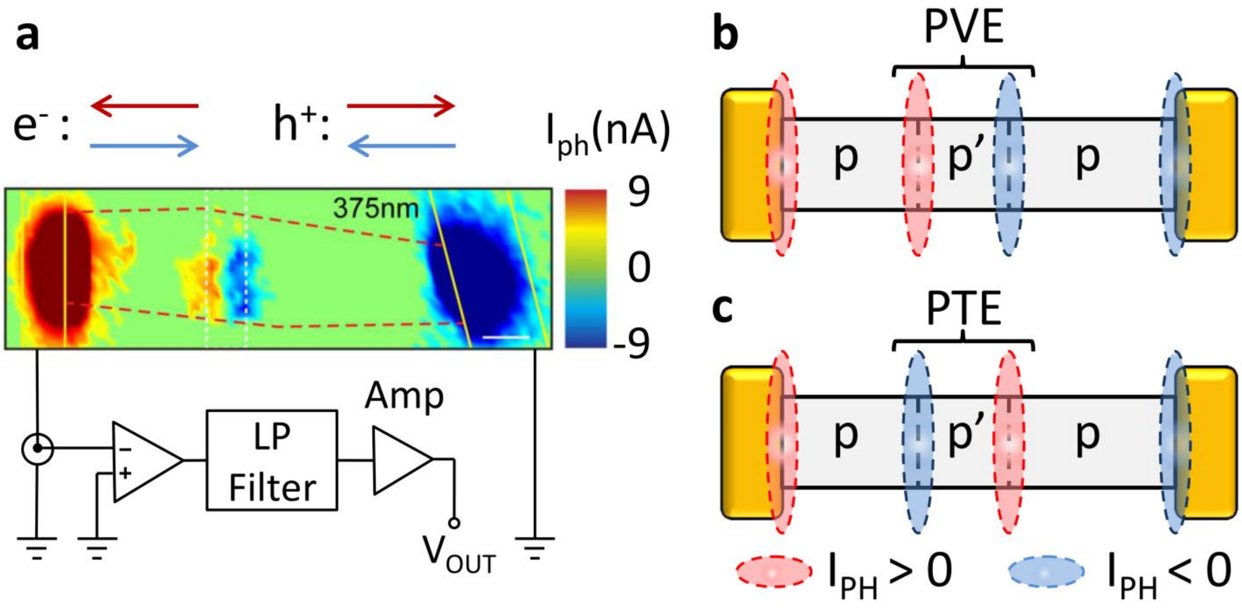

fig. S9. Direction of photocurrent at p-p′ junctions of FeCl3-FLG. (**a**) Scanning photocurrent map of a p-p'-p junction in $FeCl_3$-FLG taken from Fig. 2 (main text). Measurements were taken in short-circuit configuration with an inverting current amplifier connected to the left electrode. Positive (red) signals indicate holes drifting to the right. Two schematics of the same device illustrate the predicted direction of photocurrent local to the junctions assuming that either (**b**) the photovoltaic (PV) or (**c**) the photothermoelectric (PTE) effects is the dominant mechanism of photoresponse.

section S6. Correction of responsivity spectra for substrate reflections

The presence of a reflecting Si/SiO_2 substrate will affect the measured spectral responsivity of our $FeCl_3$-FLG photodetectors. As shown in Fig. 3c (main text), we have performed a correction which accounts for these reflections in order to examine the

intrinsic spectral response of the laser-written p-p' junctions. Figure S10a illustrates the model used for this correction which consists of an incident photon flux (Φ_0) partially absorbed by an $FeCl_3$-FLG flake of transmittance T and a transmitted remaining flux, $\Phi_t = T\Phi_0$. A portion of this transmitted flux ($\Phi_r = \Phi_t R$, where R is the reflectance of Si/SiO_2) will be reflected by the substrate and absorbed/transmitted by the $FeCl_3$-FLG, leaving a flux $\Phi_{t'} = T\Phi_r$ reflected into the environment. We neglect further reflections due to the high transmittance of $FeCl_3$-FLG and define the spectral responsivity as $\Re(\lambda) = I_{ph}/\epsilon_0\Phi$. Hence, the photon flux incident on a supported $FeCl_3$-FLG detector is effectively ($\Phi_0 + \Phi_r$) and the ratio between the measured ($\Re$) and intrinsic ($\Re_0$) responsivity may be evaluated using just T and R

$$\frac{\Re_0}{\Re} = \frac{\Phi_0}{\Phi_0 + \Phi_r} = \frac{1}{1 + TR} \quad (S34)$$

Figure S10b shows the transmittance of a four-layer $FeCl_3$-FLG sample reproduced with permission from reference (*14*) and the reflectivity of our Si/SiO_2 substrate measured in the range $420 - 700$ nm. A simulation of the substrate reflectivity using TFCalc software (Software Spectra, Inc.) shows excellent agreement with the experimental data, we therefore extrapolate the reflection coefficient from the simulated curve down to $\lambda = 375$ nm where no experimental data points are available. In the same way, we extrapolate the absorption coefficient of $FeCl_3$-FLG for the same wavelength range. The extrapolated data and the computed correction factors used in Fig. 3c (main text) are presented in table S3.

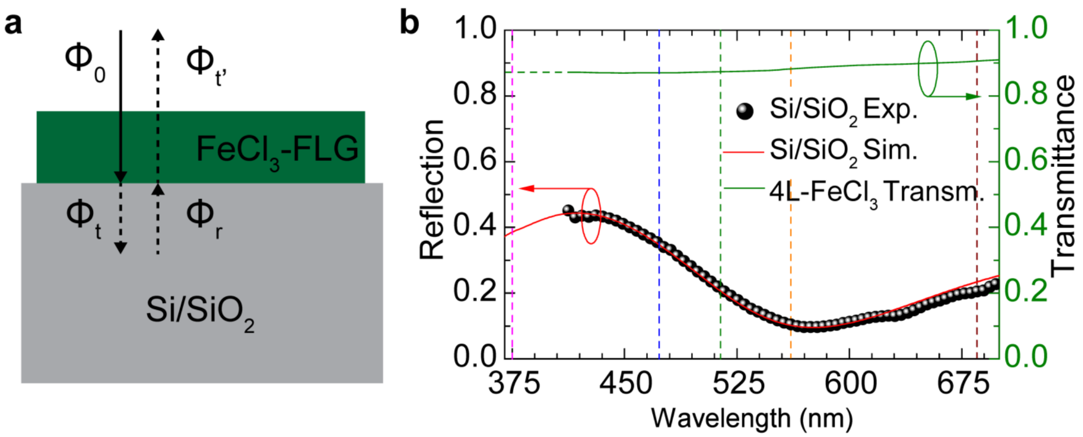

fig. S10. Correction of spectral responsivity for substrate reflections. (**a**) Concept of substrate reflection correction of responsivity: solid arrow is the incoming light (Φ_0), dotted lines represent the transmitted light through the $FeCl_3$-FLG (Φ_t) and the reflected part by the Si/SiO_2 interface (Φ_r). (**b**) Reflectivity of Silicon substrate with $290\ nm$ of SiO_2 on top: experimental values (black dots) in the region $420-700\ nm$ and computed curve (solid red line) between $370-700\ nm$; the green line represents the transmittance of 4-layer $FeCl_3$-FLG (reproduced with permission from reference (*14*)) where we extrapolated the value for the UV-A region (dotted green line). Vertical dotted lines represent the laser wavelengths used in this work.

table S3. Corrections to responsivity for the laser wavelengths used in this work.

λ (nm)	T	R	$\Re_0/\Re$
375	0.872	0.385	0.749
473	0.870	0.355	0.764
514	0.874	0.207	0.847
561	0.883	0.102	0.917
685	0.906	0.234	0.825
